# Sensing and Perception in Robotic Weeding: Innovations and Limitations for Digital Agriculture

**DOI:** 10.3390/s24206743

**Published:** 2024-10-20

**Authors:** Redmond R. Shamshiri, Abdullah Kaviani Rad, Maryam Behjati, Siva K. Balasundram

**Affiliations:** 1 Leibniz Institute for Agricultural Engineering and Bioeconomy (ATB), Max-Eyth-Allee 100, 14469 Potsdam, Germany; 2 Department of Natural Resources and Environmental Engineering, College of Agriculture, Shiraz University, Shiraz 71946-85111, Iran; arad@adaptiveagrotech.com; 3 Department of Agriculture Technology, Faculty of Agriculture, University Putra Malaysia, Serdang 43400, Selangor, Malaysia; siva@upm.edu.my

**Keywords:** agriculture, robots, digital, weed, robotic weeding

## Abstract

The challenges and drawbacks of manual weeding and herbicide usage, such as inefficiency, high costs, time-consuming tasks, and environmental pollution, have led to a shift in the agricultural industry toward digital agriculture. The utilization of advanced robotic technologies in the process of weeding serves as prominent and symbolic proof of innovations under the umbrella of digital agriculture. Typically, robotic weeding consists of three primary phases: sensing, thinking, and acting. Among these stages, sensing has considerable significance, which has resulted in the development of sophisticated sensing technology. The present study specifically examines a variety of image-based sensing systems, such as RGB, NIR, spectral, and thermal cameras. Furthermore, it discusses non-imaging systems, including lasers, seed mapping, LIDAR, ToF, and ultrasonic systems. Regarding the benefits, we can highlight the reduced expenses and zero water and soil pollution. As for the obstacles, we can point out the significant initial investment, limited precision, unfavorable environmental circumstances, as well as the scarcity of professionals and subject knowledge. This study intends to address the advantages and challenges associated with each of these sensing technologies. Moreover, the technical remarks and solutions explored in this investigation provide a straightforward framework for future studies by both scholars and administrators in the context of robotic weeding.

## 1. Introduction

The organic products market in Europe have shown substantial growth over the past decade; however, the management of weed growth in organic farming poses a significant challenge, particularly in vegetable cultivation, as the cultivated plants sometimes struggle to outcompete the naturally occurring wild plant species [1]. Weeds engage in competition with crop plants for essential resources such as water, light, and nutrients, leading to a reduction in agricultural production and crop yield [2]. The negative impact of weeds on the agricultural economy is significant, with countries like New Zealand experiencing losses of NZD 1658 million [3], India facing losses of INR 11 billion [4], and the USA and Canada suffering losses of USD and CAD 43 billion annually [5] [Table 1]. Consequently, cultivators must implement preventative measures against the growth of weeds on their plantations. Hand weeding, despite being the simplest and most commonly used technique for controlling weeds, can be labor-intensive, challenging, and, in many cases, neither cost-effective nor desirable. Additionally, it can sometimes result in crop damage. Therefore, modern conventional farming practices entail the utilization of chemicals to manage insect pests and eliminate weeds; however, the interactions between chemicals and their surrounding components are intricate, making it challenging to anticipate the outcomes in a real-world setting. Chemicals that are applied to fields may contaminate the produce that is grown there, and, more so, they persist in the soil and plants that undergo treatment. The overuse of herbicides leads to the development of weed resistance and alterations in soil microbial populations [6,7]. On the other hand, growers often lack precise knowledge of the specific types and amounts of chemical herbicides that are present in the food they cultivate and purchase from retailers. As a result, agriculture has shifted toward using fewer herbicides for weed control.

Robotic weeding offers significant advantages over traditional herbicide-based methods, particularly in terms of lower development costs and fewer regulatory complications, which is an essential point, as it underscores the potential of automation and digitalization to meet the growing demand for sustainable agricultural practices. The current trend in interest in non-chemical weed control is primarily driven by several key factors including (i) apprehension regarding the contamination of soil and surface water by herbicides, (ii) the potential risks to human health resulting from herbicide exposure or residues, (iii) the impact on flora and fauna, (iv) the emergence of herbicide resistance, and (v) the absence of approved and effective herbicides for minor crops like vegetables [8]. Hence, agricultural experts must contemplate alternate and integrated methods of weed control to decrease the application of pesticides and their effects. Currently, weed management tactics utilize weed control approaches that are broad and strong enough to effectively control the weeds. Weed management has expanded beyond hand removal or pesticide sprays and now encompasses the utilization of precise mechanical operations. Due to recent progress in digital agriculture (DA) [9], it is now feasible to employ robots for the task of “robotic weeding”, which is one of the components of automated farming. Robotic weeders have shown remarkable potential as effective weed management devices for specialty crops. They provide advantages such as lower development costs and reduced regulation compared with herbicides [10], making them a more favorable option with fewer environmental and human health hazards.

**Figure 1:**
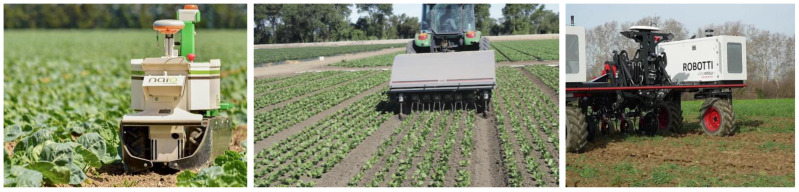
Examples of weeding robots in action performing sensing, thinking, and acting. Source of images: [28,29].

Robotic weed removal generally involves weed detection, decision-making, and action. Weed detection is achieved via utilizing accurate sensors to obtain real-time information about the presence of weeds or to create a map of weed locations for future control. Decision-making involves using the gathered information and the knowledge and expertise of the farmer to determine the appropriate course of action for weed control. The chosen weeding action is then carried out using an actuator, and the precision of the operation is evaluated to assess its effectiveness [Figure 1]. Robotic weeders have the potential to increase the range of instruments that specialty crop producers may utilize. Nevertheless, the advancement of robotic weeders necessitates a wider awareness that these devices are a feasible means of developing novel weed management instruments for specialized crops [11]. To effectively eliminate weeds inside crop rows, it is crucial for an automated system to employ dependable sensing technology that can accurately distinguish between weeds and crops at precise locations in the planting area [12]. The viability of a robotic system for controlling weeds relies on the analysis of machinery vision, the efficiency and appropriateness of the robot, the technology for applying different rates of treatment, the support system for making decisions, and the effectiveness of instruments for detecting weeds through direct or indirect means [13]. Weed detection is a crucial component of weed control since it supplies vital information for subsequent decision-making and execution processes. Accurate differentiation between weeds and crops is advantageous for weed control since the incorrect identification of weeds can lead to ineffective site-specific weed management or potentially harm the crops. Automated intra-row cultivators employ either an established crop row pattern [14] or machine vision [15] to efficiently eliminate weeds while minimizing harm to the farmed crops. Robotic weeders like the Ferrari Remoweed, Robovator, Garford In-row cultivator, and Steketee IC are able to identify the arrangement of crop rows rather than the weeds themselves and eliminate weeds that are located near the individual crop plants. Machine vision and artificial intelligence methods, including deep neural systems, are now being used to locate and recognize weeds [16]. The use of the Internet of Things (IoT) in robotic weeding enables live data monitoring and communication between sensors, machines, and the field, as well as provides information on environmental conditions, plant health, and weed growth [17]. These data can be fed into machine learning algorithms for analysis and decision-making to optimize weeding patterns, improve plant–weed differentiation, and adapt robotic systems to changing field conditions [18]. This integration enhances precision, reduces herbicide use, and contributes to more efficient and sustainable farming practices. Examples of such studies can be found in [19,20].

**Table 1:** The different types of weeds and their global distribution, as well as their characteristics.

Weed	Regional Distribution	Crops	Features	Image
*Amaranthus palmeri* (Palmer Amaranth) [21]	North America, South America	Soybeans, cotton, corn, peanuts	Glyphosate-resistant, rapid growth, prolific seed production (up to 1 million seeds per plant)	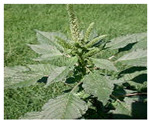
*Chenopodium album* (Lamb’s Quarters) [22]	North America, Europe, Asia	Maize, soybeans, vegetables	Highly competitive, high seed production, wide tolerance to different environments	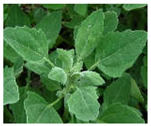
*Convolvulus arvensis* (Field Bindweed) [23]	Worldwide (temperate regions)	Wheat, barley, corn, cotton	Deep root system, difficult to control, produces many seeds (seeds can remain viable for years)	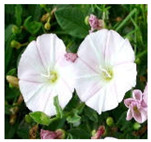
*Cyperus rotundus* (Purple Nutsedge) [24]	Tropics, subtropics, temperate zones	Rice, sugarcane, vegetables	Perennial weed, forms tubers, very competitive, difficult to eradicate	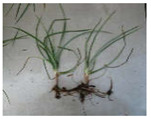
*Echinochloa crus-galli* (Barnyardgrass) [25]	Worldwide (tropics and subtropics)	Rice, maize, wheat	Highly adaptive, competitive, rapid growth, herbicide-resistant populations	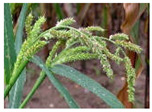
*Sorghum halepense* (Johnsongrass) [26]	North America, Europe, Asia, Africa	Corn, soybeans, sorghum, cotton	Perennial, spreads via rhizomes and seeds, highly competitive	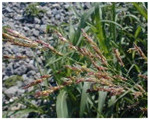
*Setaria viridis* (Green Foxtail) [27]	Worldwide	Cereals (wheat, barley, oats), corn	Annual grass, rapid seed germination, competitive with crops for nutrients	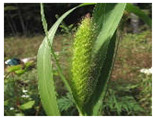

Although robotic weeding systems have undergone significant improvements in the last 30 years, several existing problems still need to be addressed for widespread adoption, including weed identification accuracy (particularly in densely vegetated areas) and weed detection under varying light conditions (such as shadows and direct sunlight). Differentiating between crops and weeds in such environments remains difficult due to the similar visual characteristics of some species, which remains problematic for many current systems. We explored key technologies, including imaging and non-imaging solutions such as RGB-D cameras, LIDAR, time-of-flight (ToF) sensors, multispectral and hyperspectral imaging, as well as ultrasonic and radar-based solutions, and assess their current capabilities and limitations in identifying and targeting weeds. The presented materials aim to guide researchers in understanding the state of these sensing technologies and their role in enhancing robotic weeding systems while also highlighting the areas requiring further development to shape the future of precision agriculture.

## 2. Innovation in Sensing System for Robotic Weeding

Weed distribution and spatial heterogeneity within farms can be detected via employing satellite imagery for a general overview, while near-ground approaches can be used for applications in real time that require more precise sensing processes [30]. Remotely sensed aerial spectrum scans may be applied to create prescription maps in geo-statistical programs, allowing for the adjustment of pesticide treatment rates based on the exact location of the weeds. Nevertheless, these systems primarily depend on high-quality and up-to-date aerial data, as well as thorough data management and processing, to detect weed areas. As a result, they are not suited for real-time field applications [31]. Therefore, research on robotic sensors is progressing toward the precise ground-based identification and differentiation of weeds via analyzing plant characteristics.

Plant features may be accurately evaluated for species recognition and targeted action through analyzing visuals captured at certain wavelengths, employing computer vision, or via analyzing 3D point clouds obtained from laser scanners or 3D cameras. Some examples of these traits include the quantification of fruit quantity in specific areas of a tree’s canopy (for purposes such as harvesting or thinning), the characteristics of the tree’s trunk and branches in terms of their geometry and structure (for tasks like pruning), the shape of the tree (useful for identifying and categorizing weeds), and the assessment of plant stress based on images of leaves and stems (for targeted spraying or fertilizing). Modern sensing technologies offer higher levels of detail and accuracy in terms of both space and time [32,33,34,35] and are divided into two categories: imaging sensors, including smart cameras, and non-imaging sensors, such as lasers and ultrasonic systems.

A smart camera collects valuable data, uses complex algorithms, and renders informed judgments for particular purposes, such as automated operation [36]. The utilization of smart cameras was initiated in the 1990s across several industrial sectors, and currently they have been extensively researched for their applicability in agriculture due to their non-invasive nature, rapidity, efficiency, and cost-effectiveness, all of which contribute to a reduction in labor-intensive tasks. The cameras may be used with various kinds of algorithms for image processing to leverage the color, structure, and textural data included in the captured images. These algorithms are capable of examining various characteristics associated with plant and weed canopies, as well as providing information on bare soil. In addition, such techniques are unaffected by geographical mistakes and do not require additional pre-processing or the generation of prescription maps, unlike remotely sensed data [37]. Optoelectronic sensors typically assess reflection intensities in a limited number of spectral bands, generally one or two, specifically in the red/near-infrared (R/NIR) range. They demonstrate high levels of timeliness, effectiveness, and cost-efficiency in distinguishing plants from the environment (soil) and are being successfully introduced to the market. WeedSeeker^®^, GreenSeeker^®^ (manufactured by Trimble Agriculture, Sunnyvale, CA, USA), and WEED-it (Rometron, Steenderen, The Netherlands) are widely used pieces of equipment for sensing vegetation in the commercial sector. In the case studies that employed WeedSeeker^®^, sugarcane herbicide reductions of over 80% were reported [38], and it is compatible with various crops, including corn, soybeans, wheat, and specialized settings like vineyards and orchards. Riczu and Tamás [39] conducted a study in an apple orchard that employed a Tetracam ADC multispectral camera and a GreenSeeker^®^ 505 vegetation index meter to detect weeds. The study revealed a high correlation between weed coverage and vegetation indices. In addition, Kool et al. [40] investigated the ability of the Rometron WEED-IT sensor to identify weeds in a soybean field and pointed out that the WEED-IT sensor can distinguish between living and non-living plant material but cannot differentiate between weeds and crops. Table 2 summarizes some of the commercialized weeding robots and their features and benefits.

**Figure 2:**
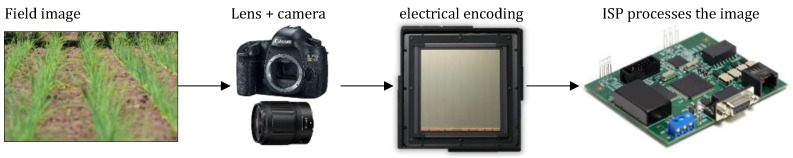
Principle of a camera-based weed detection sensor. Adapted from [49,50].

The camera-based sensor comprises three primary components: (a) an integrated device, based on a charge-coupled device (CCD), that includes its electrical components and connectors for powering and connectivity to the operating system, all enclosed within a casing; (b) an optical lens; and (c) an ultraviolet (UV) and infrared cut filter, which regulates the intake of specific wavelengths [48]. Figure 2 depicts the components arranged together as a whole unit and individually separated. Smart cameras have the capability to reduce the usage of agrochemicals, conduct tasks without causing any harm or damage, decrease expenses, and minimize the amount of manual work required for sorting.

In addition to the ground sensors, the application of drones or unmanned aerial vehicles (UAVs) in robotic weeding has become increasingly significant in digital agriculture. UAVs equipped with advanced imaging technologies, such as RGB, multispectral, hyperspectral, and thermal cameras, can provide real-time aerial high-resolution data on weed distribution, plant health, and field conditions, enabling the precise mapping of weed-infested areas, which can be fed into robotic weeding systems for targeted action. UAVs can cover large agricultural areas quickly and efficiently, reducing the need for manual field surveys and improving overall decision-making in weed management. By integrating UAVs with machine learning algorithms, the aerial data can be analyzed to differentiate between crops and weeds, optimize robotic weeding patterns, and reduce the use of herbicides. Additionally, UAVs can be used for monitoring the effectiveness of robotic weeding operations, ensuring that weeds are accurately removed while minimizing crop damage. This interaction between aerial and ground-based robotics enhances precision, reduces labor costs, and promotes more sustainable agricultural practices.

## 3. Imaging Solutions

Imaging sensors, which utilize a range of imaging technologies including RGB cameras, near-infrared, and hyperspectral imagery, are crucial for achieving accurate and focused weed management while reducing the need for pesticides and laborious tasks. An important benefit of image sensors in robotic weeding is their capacity to offer instantaneous information on the distribution of crops and weeds in the cultivation area. Through the acquisition of intricate field pictures, these sensors enable precise discrimination and categorization of crops and weeds with exceptional precision [51]. Subsequently, algorithms powered by machine learning analyze these data to identify certain weed species and determine the most suitable responses, such as selectively applying pesticides or physically removing the weeds [52]. Utilizing cameras in computerized weeding machines presents distinct advantages compared with conventional weed management approaches [53]. First and foremost, the capacity to accurately focus on eliminating weeds while protecting crops leads to substantial financial savings and improved productivity. By selectively spraying herbicides exclusively in areas where weeds are prevalent, farmers may decrease their pesticide consumption, resulting in monetary benefits and limiting the ecological consequences of farming.

RGB cameras are widely used due to their cost-effectiveness and ability to capture high-resolution color images in the visible spectrum, making them suitable for basic plant differentiation tasks. However, they are limited in detecting plant stress or characteristics beyond the visible range. Near-infrared (NIR) cameras, on the other hand, are better in detecting chlorophyll levels and plant water content, but they are more expensive and lack the color differentiation of RGB cameras. Spectral and multispectral cameras go a step further, capturing data across multiple wavelengths, allowing for an advanced analysis of plant characteristics such as nutrient deficiencies or disease, making them ideal for precision agriculture. However, their high cost and the complexity of processing large datasets make them less accessible for real-time applications. Thermal cameras detect heat signatures, enabling the identification of plant stress and moisture levels, and they perform well in low light or nighttime conditions, although thermal imaging alone may not provide enough detail for accurate plant–weed differentiation and is sensitive to environmental factors such as wind and surface reflectivity. Therefore, each camera type presents a trade-off between cost, data richness, and the complexity of integration, and their selection depends on the specific requirements of the robotic weeding system. It should be noted that the independent functionality of robotic weeding systems coupled with image sensors enables continuous weed surveillance and control, guaranteeing prompt interventions and minimizing the likelihood of weed infection. The following are discussions of the most prevalent sensor systems mounted on weeding robots.

### 3.1. RGB Cameras

**Figure 3:**
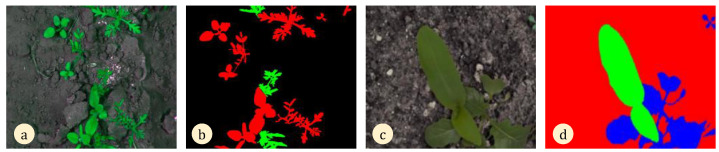
The identification and differentiation of crops and weeds, showing (**a**,**b**) the difference between sugarcane (green), soil (black), and weeds (red), and (**c**,**d**) the differences between classified weeds (blue) and corn (green) using the RGB camera. Adapted from [75,76].

Accurate data at different stages of plant growth are necessary to accurately identify weeds via phenotyping diverse plant properties [54,55], and computer vision technology, in this context, empowers machines to autonomously carry out a range of tasks, such as planting and harvesting, which has garnered significant interest in recent times [56]. To automate the detection of weeds and distinguish them from crops, tagged photos should be used to train models. In this regard, Sudars et al. [57] used three Red-Green-Blue (RGB) digital cameras, including the Canon EOS 800D (manufactured by Canon, Tokyo, Japan), Intel Real-Sense D435 (Intel, Santa Clara, CA, USA), and Sony W800 (Sony, Tokyo, Japan), to capture annotated pictures. RGB may offer geometric data in color pictures and 3D forms [58,59], together with per-pixel depth, making it highly valuable in the field of robotics due to its affordable cost [60]. Putra and Soni [61] suggested the use of RGB digital cameras as a cost-effective tool to evaluate plant biophysical parameters, such as nitrogen levels. Costa et al. [62] employed a drone-mounted RGB camera and a genetic algorithm to develop a novel visible index for estimating the normalized difference vegetation index (NDVI) in citrus, grapes, and sugarcane. They concluded that using an RGB camera was a financially efficient alternative to remote sensing, with a mean average error of 0.052 and a percentage error of 6.89%. Burkart et al. [54] successfully computed the green-red-vegetation index (GRVI) for barley via utilizing RGB photographs, thereby establishing the efficacy of RGB for measuring information relevant to farm management. According to research conducted by Gracia-Romero et al. [63], the RGB index demonstrated superior accuracy compared with multispectral methods in assessing maize yield. Shoot weight was substantially correlated with NDVI estimation (R^2^ = 0.69) in viticulture using a high-resolution RGB camera, according to Matese and Gennaro [64]. De Lima et al. [65] devised a technique to estimate the NIR band via utilizing RGB photographs. This method allows for the assessment of crop characteristics and vegetation index with an error rate of less than 9%. Endres et al. [66] showed that the Microsoft Kinect RGB-D camera is capable of overcoming the difficulties posed by quick camera motions and environments with few distinctive features. Additionally, it offers a speedy online operation capability. Based on the literature, robotic plant detection might be advantageous for preemptively eradicating weeds prior to growing crops. Marin et al. [67] and Gasparovic et al. [68] employed a drone equipped with a 1.5-megapixel RGB camera to identify weeds, since it is more economically viable compared with multispectral cameras. In an investigation carried out by Rasmussen et al. [69], the pictures captured by the RGB camera were separated into 1m-2 segments based on the altitude of the aircraft. This approach enabled the identification of weeds inside the crop area. Huang et al. [60] demonstrated that drones carrying RGB-NIR cameras have the capability of determining weed species that are resistant to the glyphosate herbicide. An affordable RGB image sensor is capable of detecting weed patches in fields at heights ranging from 20 to 120 m [70]. Figure 3 shows the vegetation pixel categorization of two test photos for weed detection and identification tasks. The classification is color-coded.

Although RGB cameras have many benefits in recognizing weeds, recent studies have focused on the difficulties of using RGB cameras to assess plant parameters such as tree shade, inadequate sunlight, the existence of shoots and leaves, adverse environmental conditions, and time limitations [61,71,72]. Moreover, the lack of homogeneity might impair the functionality of the detecting mechanism [59]. Several plant identification experiments have conducted the extraction and analysis of morphological characteristics of either a leaf or an entire plant, including measurements such as length, breadth, perimeter dimensions, roundness, circularity, convexity, and moment. In their study, Slaughter et al. [73] examined various systems and determined that they typically exhibited excellent recognition accuracy in optimal circumstances, when there were no obstructions to the leaf or plant and the leaf was intact. Nevertheless, they lacked resilience when it came to the shadowing issue produced by visual flaws of the plant, such as damage by insects or wind distortion, which are frequently observed in real situations. Lottes et al. [74] additionally pointed out the problem of occlusion, which occurs when weeds are partially concealed by crops or other vegetation, resulting in instances when they are not detected.

### 3.2. NIR Cameras

**Figure 4:**
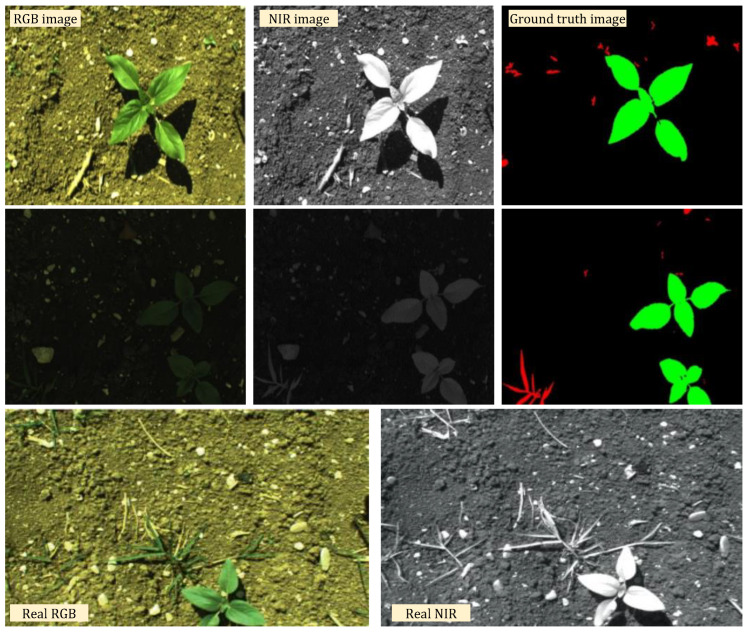
An example of scenes from the field and sunflower-weed segmentation using RGB and NIR. Reprinted from [90,91].

NIR radiation was primarily recorded using infrared (IR)-sensitive plates or film emulsions [77]. Employing a NIR camera becomes highly efficacious in surmounting the obstacle posed by inadequate illumination in farming environments. Under conditions of limited illumination, imaging systems capture color RGB pictures that are adversely affected by significant noise, resulting in the degradation of both color accuracy and texture details. Hence, NIR photographs have the benefit of disregarding external light interference, allowing them to capture imperceptible information that is unattainable for conventional RGB cameras [78]. Consumer-grade digital cameras, whether equipped with external filters or not, are generally recognized as a cost-efficient method for vegetation monitoring. These cameras have the power to produce time-series data on the biophysical traits of plants [79]. Sakamoto et al. [80] investigated the possible usefulness of an economical camera observation system known as the crop phenology recording system (CPRS) as a substitute method for monitoring the seasonal progression of crop development. The CPRS, which comprised two compact digital cameras, was employed to continuously acquire visible and NIR images of maize in 2009 and soybean in 2010 for an hour, spanning both daytime and night. Zhang et al. [81] conducted a study on image classification and accuracy assessment which found that the inclusion of supplementary NIR band imagery in a cropping area in Texas, USA, resulted in enhanced accuracy in crop classification compared with the RGB imagery. An investigation was conducted into the effectiveness of NIR spectroscopy as a non-invasive method for estimating avocado maturity and, consequently, ingesting quality, using the dry matter content of whole, intact fruit as the primary criterion [82] [Figure 4].

Most remote and proximal sensor instruments are founded on NIR spectroscopy and are described as environmentally friendly, non-invasive, and user-friendly [83]. Zhu et al. [84] introduced a novel approach that utilizes two NIR digital cameras for the precise and rapid measurement of water turbidity. The comparison with a commercial turbidimeter demonstrated that this technique has a high level of accuracy in determining standard solutions throughout a broader linear range. Furthermore, the findings obtained via measuring real samples using this method were similar to those obtained from the turbidimeter, confirming the practicality of this approach. Hunt et al. [85] conducted tests using a Near-Infrared-Green-Blue digital camera mounted on an unmanned aerial vehicle (UAV) system. The tests were carried out on two fields of winter wheat that had different levels of fertilizer application. They revealed a significant correlation between the leaf area index (LAI) and the green normalized difference vegetation index (GNDVI). A benefit of this affordable NIR-Green-Blue digital camera is the ability to immediately analyze photographs from the camera itself [86]. Spectral imaging, although capable of unveiling the intricacies of our surroundings, is presently limited to laboratories due to the requirement for intricate and expensive equipment [87]. Although there have been some positive outcomes, these digital camera systems typically experience signal aberrations caused by the image processing systems on board, resulting in a limited capacity to acquire quantitative data [88]. The issue of color distortion arises from the sensors’ sensitivity to the overlapping of visible and near-infrared spectral bands [89]. Often, weeds and crops exhibit similar spectral properties in the NIR range, making them difficult to distinguish. Keeping NIR sensors calibrated regularly is crucial to ensuring accuracy, especially in variable lighting conditions, which can be labor-intensive, and a significant effect of exposed soil on NIR reflectance values can result in an inaccurate detection of weeds.

### 3.3. Spectral, Hyperspectral, and Multispectral Cameras

Historically, conventional satellite systems like the U.S. Landsat satellites and the French SPOT satellites have been utilized to observe agricultural growth conditions and approximate crop production across extensive geographical regions. Nevertheless, NIR spectroscopy has restricted applicability when it comes to evaluating variations in crop output within a field due to its low level of detail in terms of spatial resolution, infrequent data collection intervals, and delayed data transmission [92]. In the context of the weed issue, prior research has demonstrated encouraging outcomes using either color cameras or spectrum imaging techniques for plant recognition and categorization. Nonetheless, conventional color camera systems have encountered difficulties in handling the issue of overlapped leaves [93]. Conventional imaging using RGB and/or NIR sensors does not possess the full range of wavelengths and accuracy required to analyze the characteristics of substances and organisms that can only be achieved by hyperspectral sensors [94]. Hyperspectral imaging, also known as imaging spectroscopy, integrates computer vision technology with optical spectroscopy [95], originally used for remote-sensing purposes, and has evolved to enable comprehensive and dependable analysis of both intrinsic properties and external features of samples [96]. The hyperspectral camera has the capability of capturing landscape photographs that encompass crops, weeds, and the soil surface, and it can offer more detailed data compared with ordinary RGB photos [97]. A hyperspectral picture is composed of a substantial quantity of pixel spectra. If the pixel spectrum was determined to be of plant origin, it was classified as either a crop or a weed.

In the last 15 years, there has been a significant growth in the study, development, and utilization of hyperspectral imaging in the food and farming industries [98]. The literature over the past 15 years has repeatedly shown that spectral vegetation fingerprints may be used to differentiate between crop and weed species [99]. The efficacy of spectral imaging in separating vegetation from soil [100,101] and distinguishing agricultural plants from weeds has been documented in several studies [102]. In a study by Okamoto et al. [103], hyperspectral images were used. The photographs included cultivated plants, weeds, and soil. Initially, the pixels representing the plant (either crop or weed) were isolated from the surrounding soil surface. This method leveraged the disparity in spectral patterns between plants and soil. Subsequently, the image pixels of the sugar beet crop and four kinds of weeds were categorized based on the investigation of the disparity in spectral properties among the different varieties of plants. The categorized items were obtained via utilizing wavelet transforms for the purposes of data compression, reducing noise, and extracting features. Subsequently, stepwise discriminant analysis was performed. The test yielded a success rate of around 80% in plant categorization. Furthermore, multispectral imaging is now being utilized in this field [104]. Feyaerts and van Gool [105] introduced an internet-based system that differentiates between crops and weeds using multispectral reflectance data collected with an imaging spectrograph. During field experiments, it was shown that 86% of the plant tests (80% of crop samples and 91% of weed samples) showed a drop in herbicide application of up to 90%. In a study by Slaughter et al. [106], using multispectral images of leaf reflectance ranging from 384 to 810 nm, a site-specific classifier was developed to distinguish lettuce plants from weeds in California’s direct-sown fields. Based on an analysis of over 7000 individual spectra representing 150 plants, an average accuracy of 90.3% was obtained for crop identification versus weed identification. Based on the results obtained by Yu et al. [107] using multispectral imaging UAVs in rice fields, the effectiveness of the weed identification was 93.47%. Moreover, multispectral imaging is utilized in various fields like the assessment of plant water status, plant nutrient levels and illnesses, insect-induced damage, weed identification, evaluation of fruit quality, determination of the quantity of mature and immature fruits, and assessment of the fruit maturity state [108].

**Figure 5:**
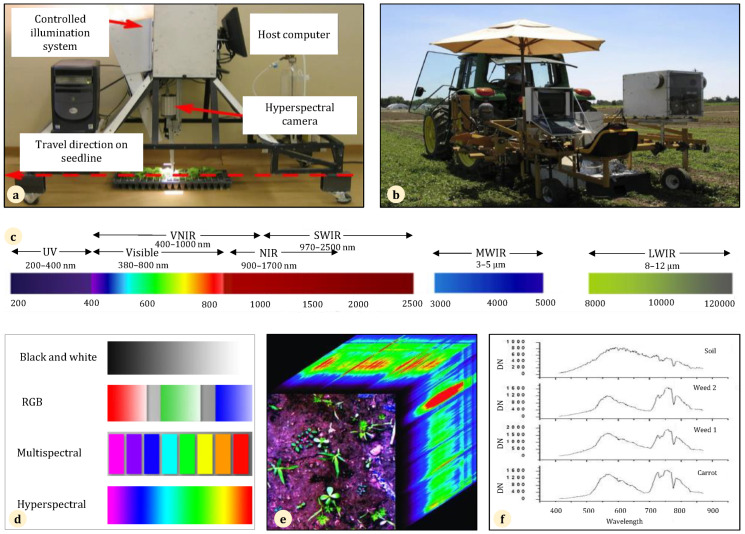
(**a**) Main components of a hyperspectral imaging system and (**b**) configuration during on-site data collection, reprinted from [111]; (**c**) wavelength regions for hyperspectral imaging; (**d**) different modes of imaging illustrating the benefits of hyperspectral imaging, reprinted from [112]; (**e**) the sample spectral data; (**f**) typical spectra of several surface objects (soil, weeds, and crops), reprinted from [113].

The spectrum reflectance of plants is influenced by the cellular and biochemical composition of the leaves, as well as the arrangement of leaves in the canopy. Vrindts et al. [109] employed the reflectance spectra of sugar beet and weed canopies to assess the potential for weed identification. During field testing, a maximum of 95% of the sugar beets were accurately identified as sugar beets, whereas up to 84% of the weeds were correctly labeled as weeds. Lee et al. [110] conducted research where they created a sophisticated robotic system capable of identifying and targeting specific weeds in real-time. This system utilized advanced machine vision technology and precise chemical administration methods to selectively apply herbicides to the weeds found within crop rows. The robotic vision system had a processing time of 0.34 s per picture. Each image represented a patch of seedlings measuring 11.43 cm by 10.16 cm and contained 10 plant objects. This allowed the prototype robotic weed management system to travel at a constant speed of 1.20 km per hour [Figure 5].

A number of studies that utilized spectral characteristics to differentiate crop plants from weeds were reviewed by Zwiggelaar [114]. He pointed out that while employing spectral cues to differentiate between certain groups of weed and crop plants is successful, the spectral wavebands chosen for classification often vary depending on the specific weed and crop combinations. Within an actual-world agricultural environment, there exists a multitude of weed species, which presents a challenge in selecting appropriate wavebands and designing algorithms to accurately differentiate between crop plants and distinct varieties of weeds. The gathering of hyperspectral data relies on several factors, including the range of wavelengths in the electromagnetic spectrum, the method of imaging, and the imaging device [115]. Additionally, being a passive sensor that is influenced by external conditions, such as changes in sunshine intensity, it is not considered to be a dependable method for classifying plant species [116,117,118]. The application of stereovision for corn plant identification [119] and structural analysis has been documented [120]. The primary obstacle to employing stereovision in real agricultural systems is the difficulty in finding corresponding points due to the absence of leaf texture, the intricate nature of the canopy structure, occlusion, and variations in illumination conditions [121]. The rates of the achievement of plant categorization are contingent upon the crop’s condition, the quantity of wavelengths, and the gadget’s location and spectral filtration capabilities [122]. There is a risk that narrow-wavelength coverage may not capture all of the information necessary to distinguish between weeds and crops, especially when their spectral characteristics are similar. It is also necessary to calibrate sensors frequently in order to maintain accuracy, particularly in field conditions that vary widely, thereby making the process labor-intensive. Moreover, in their study, Andujar and Martinez-Guanter [123] discussed the difficulties of spectral sensing, especially its sensitivity to light intensity and soil moisture. Consequently, aspects such as calibration accuracy, data analysis complexity, and environmental factors influence the effectiveness of spectral sensing. It is possible for their accuracy to be limited as a result of inconsistencies in feature extraction and calibration issues [124].

Currently, there are other spectral indices available for different precision agricultural purposes instead of just relying on normalized difference vegetation indices [32]. NIR-Hyperspectral is a novel method that integrates traditional NIR spectroscopy and imaging techniques to acquire both spectral and spatial data from a field or sample at the same time. As a type of hyperspectral sensing that specifically targets the NIR region of the electromagnetic spectrum, NIR-Hyperspectral usually spans wavelengths between 700 nm and 2500 nm and focuses specifically on the near-infrared region of the electromagnetic spectrum, a region that is particularly useful for analyzing vegetation. The procedure is non-invasive, environmentally friendly, rapid, and reasonably cost-effective for each analysis [125].

A comparison of spectral, hyperspectral, NIR-Hyperspectral, and multispectral sensing is presented in Table 3. Because of restricted availability beyond the field of science, hyperspectral photos have not been extensively utilized in precision farming. Recently, there have been advancements in the development of small and affordable airborne hyperspectral sensors, such as the Headwall Micro-Hyperspec and Cubert UHD 185-Firefly. Additionally, advanced hyperspectral sensors for space use, such as PRISMA, DESIS, EnMAP, and HyspIRI, have either been flown or are planned to be launched. Agricultural applications are increasingly gaining access to hyperspectral imaging technology. However, the gathering, processing, and analysis of hyperspectral images continue to pose challenges in research due to factors such as the vast volume of data, the high dimensionality of the data, and the complexity of information analysis [126].

### 3.4. Thermal Cameras

Image sensor-based non-contact physiological measures have experienced significant advancements in recent years. Thermal cameras have the benefit of being able to determine temperature in the absence of light, along with the potential to be used for physiological measurements. Several studies have utilized thermal cameras to quantify physiological data, including respiration rate, pulse, and body temperature [130]. Leira et al. [131] conducted research where they evaluated a thermal imaging system using thermal video data from a trial flight. The results showed that the system was able to recognize 99.6% of items of interest situated on the ocean surface. Out of all the items that were identified, just a mere 5% turned out to be false positives. Moreover, it accurately identified 93.3% of the object categories it has been trained to categorize. The classifier is quite flexible, enabling the user to promptly specify the object attributes to be taken into account during classification as well as the sorts of objects to be classified.

The use of thermal photography has been acknowledged as a relatively cost-efficient method for identifying the exact position of different varieties of weeds in the field [132]. Thermal imaging can also forecast crop yield under different water levels as well as predict the viability of dormant seeds after the seeds have absorbed water via monitoring changes in temperature [133]. Weed mapping has traditionally relied on spectral, textural, and structural observations, with little attention given to thermal metrics such as canopy temperature (CT). Thermal monitoring can enhance the precision of weed mapping when combined with other remote-sensing measures in a data-fusion context. The highest performance for weed mapping was attained via incorporating textural, structural, and thermal information [134]. The study carried out by Shirzadifar et al. [135] aimed to confirm the usefulness of superior-resolution multispectral and thermal UAS pictures in identifying different types of weeds and glyphosate-resistant weeds during the early phases of their growth. An empirical study was carried out to assess supervised classification techniques for the identification of three weed species, namely waterhemp (*Amaranthus rudis*), kochia (*Kochia scoparia*), and ragweed (*Ambrosia artemisiifolia* L.). The categorization of vulnerable and resistant weeds based on canopy temperature resulted in discriminating accuracies of 88%, 93%, and 92% for glyphosate-resistant kochia, waterhemp, and ragweed, respectively.

**Figure 6:**
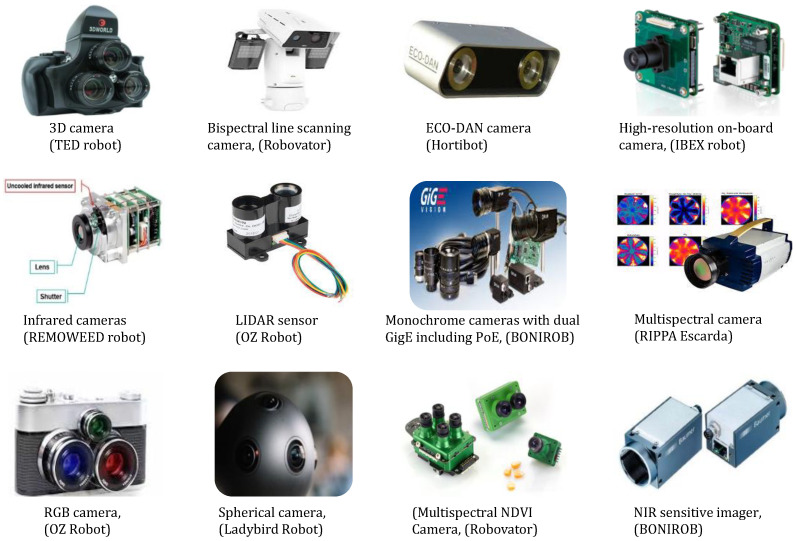
Several examples of cameras applied to the weeding robots.

UAV-assisted thermal and multispectral imagery gathering has the capability to recognize the biophysical features of different types of weeds throughout the period of growth. This includes the ability to distinguish between groups of weeds that are susceptible or resistant to glyphosate based on their canopy temperature and the use of advanced weed identification computational methods powered by deep learning. The inconsistent results obtained from thermal imaging indicated that canopy temperature information is an unreliable indicator of glyphosate resistance. An assessment should be conducted to investigate the efficacy of alternative multispectral imaging methods or analytic models in order to enhance the accuracy of identifying glyphosate-resistant weed species [136]. Thermography, when used in conjunction with other camera sensors and data-mining methods, plays a vital role in the adoption of a more automated, accurate, and environmentally friendly approach to agriculture. Nevertheless, thermal data require adjustments that account for the ambient and measurement circumstances to ensure an accurate interpretation of the data [137]. Under varying conditions, temperatures and solar radiation can cause inconsistencies in thermal signatures, making it difficult to identify weeds from crops. The differences in moisture content between weeds and crops also affected thermal contrast, resulting in a decrease in weed detection accuracy. It is difficult to distinguish crops from weeds, particularly when their thermal signatures are similar. In the presence of mixed weeds, the detection process is complicated, which results in a higher number of false detections. Other challenges associated with thermal sensing include problems related to real-time data processing and integration with other imaging modalities. Figure 6 shows several examples of traditional cameras in the field of robotic weeding.

## 4. Non-Imaging Solutions

In the past thirty years, there has been a rise in the development of near-range sensor technologies specifically designed to be installed on vehicles for use in precision agriculture (PA) applications. These innovations primarily aim to recognize vegetation and assess its physiological condition via analyzing its spectral and morphological features. The most utilized sensors in PA applications are cameras, spectrometers, fluorometers, and distance sensors [138]. The primary challenge in developing effective site-specific weed control systems is the automated identification and detection of weeds in agricultural fields. The research progress is outlined for two distinct methods of addressing the issue: distant sensing weed mapping and ground-based detection via applying digital cameras or non-imaging sensors. A diverse range of sensors, such as global navigation satellite systems (GNSS), lasers, and ultrasonic systems, are available to enhance the effectiveness of weed management when used in conjunction with mechanical systems [139]. Aerial imagery has proven effective in identifying specific weed patches that are densely and uniformly distributed and possess distinctive spectral properties. The process of identifying weeds is hindered due to the blending of different spectral signals inside the relatively large pixels, which are usually greater than 1 by 1 m. Consequently, it is not feasible to identify weeds from images when the weed seedlings are scattered among the crop plants [140].

The presence of overlapping vegetation poses a barrier to accurately detecting weeds. Crop-plant signaling refers to a novel method of interaction between robots and plants. It enables the visibility of external fluorescent signals that are applied to crop plants with the purpose of identifying crops and weeds [12]. Raja et al. [141] conducted research where they built a weed-knife control system for tomato and lettuce crops. This system utilized robot vision and a unique 3D geometric identification algorithm to automate weed management. The technology effectively recognized the crop signal from obstructed crop plants while moving at speeds of up to 3.2 km/h. The field trials demonstrated that the system has the capability to decrease the quantity of weed plants by 83% in the seedling zone. The accuracy of crop detection was quantified at 97.8%, with a precision of 0.998 and a recall of 0.952. The detection process took 30 milliseconds for each frame. Nanni et al. [142] examined the effectiveness of airborne hyperspectral imaging and non-imaging sensors in the VIS–NIR–SWIR spectral range for determining particle size and soil organic matter (SOM) in the top layer of tropical soils. At the prediction stage, the non-imaging sensor yielded R^2^ values above 0.7 for clay and SOM, whereas the R^2^ value for sand was below 0.54. The image sensor produced models for clay, sand, and SOM with R^2^ values of 0.62, 0.66, and 0.67, respectively. The investigation conducted by Åstrand and Baerveldt [143] has shown that the row-recognition system, utilizing an innovative algorithm, can effectively navigate the robot with a precision of ±2 cm. In another investigation carried out by Klose et al. [144], a robot was capable of independently navigating through cornfields. The robot was outfitted with sensor systems and an actuator that allowed it to carry out weed control tasks among the rows of corn.

Non-visual sensing on farms faced obstacles such as excessively repetitious landscapes, reflection, and burnt pictures due to bright sunshine and rugged terrain, despite the numerous advantages of non-imaging sensors in the weeding process [145]. It is crucial to optimize and enhance agricultural robotics via developing quicker processing algorithms, improving communication between automation platforms and executions, and implementing sophisticated sensor systems [146]. Atefi et al. [147] had a positive outlook on the future advancements of autonomous and robotic systems in the field of plant phenotyping research. They predicted significant progress in the next decade which would propel the study into a new age. Non-imaging sensors provide measurements of a specific location in the field. The subsequent methods for detecting weeds without using imaging techniques might be categorized as follows: The initial stage is the identification of plant species via the examination of their spectral features, which involves the detection of reflecting or emitting light utilizing spectroscopy instruments and fluorescence detectors. The second phase examines characteristics such as the elevation above the ground, utilizing advanced technology such as light detection and ranging (LIDAR) and ultrasonic equipment [138].

### 4.1. Laser-Driven Sensing

Despite the limited knowledge regarding the efficacy of lasers in managing weed seeds [148], the use of tiny self-navigating robots that carry lasers has the potential to enhance sustainable and environmentally friendly weed control methods. These robots could either replace or complement the use of herbicides and manual weed measures [149]. Edith Cowan University (ECU) in Perth, Australia, is currently developing a weed sensor and spraying system that has the potential to reduce pesticide expenses. The new technology will enhance crop spraying via providing an unparalleled degree of precision and effectiveness since it can identify and target certain weeds exclusively. A bespoke combination module is used to direct three laser beams with collimated and evenly polarized properties, each having distinct wavelengths, down a single optical channel. Subsequently, every outgoing beam passes through an optical structure that generates a laser spot array characterized by precise spatial precision. Every outgoing beam is aligned and parallel. This methodology is advantageous due to the fact that all three wavelength-specific spot arrays leave a comparable optical imprint on the surface of the vegetation. Consequently, every intensity reading correlates to the identical geographic location on the soil, stem, or leaf. Furthermore, when an array of collimated laser diode beams with a Gaussian profile is projected, it results in a significantly larger and uniform concentration of intensity across the radiation pattern, in contrast to LED lighting. This study presented the initial documented design of a sensor system that utilizes laser light spectroscopy for the purpose of classifying plants and soil.

**Figure 7:**
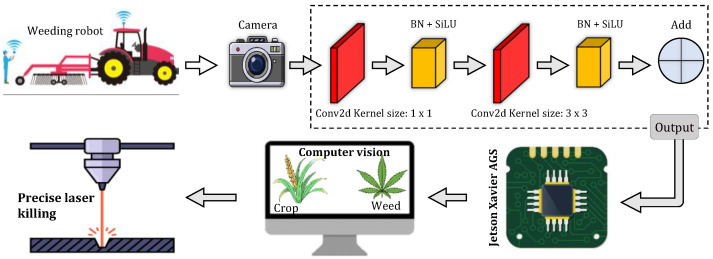
Performance of a laser-powered device for identifying and locating weeds. Reprinted from [154].

During this phase of the sensor’s advancement, the primary hardware components are assembled into a unified housing unit that is connected to a personal computer (PC). The design comprises a laser combination module, an optical structure for generating multiple beams, a collecting lens, and a charged coupled device (CCD) image sensor. The software governs the order in which lasers are activated and collects data from the image sensor. Discrimination is accomplished via measuring and contrasting the reflectance qualities of plants at different wavelengths. The laser module consists of three laser diodes with distinct wavelengths, which are precisely aligned with two free-space beam combiners. This generates three laser beams that are collimated, overlapped, and have the same polarization angle. The three beams, when merged, go through a single aperture in the module. An optical device known as a “black box” has been created to generate several laser spots with customizable spacing. The structure has the capability to produce a maximum of 14 spots from a single laser beam that emerges from the beam combiner. There is a spatial resolution of 15 mm between the points. This precise spacing enables the identification of plants with extremely slender stems or leaves. A 1024-pixel image sensor with a pixel size of 14 × 14 µm is employed to measure the light intensities reflected from various spots. Once the intended wavelength of a particular laser is activated, the image sensor captures a single frame that displays the Gaussian intensity profile of the spot array landing on the plant or soil being examined. The maximum intensity value of each area is retrieved and utilized for computing the spectral properties. If the optical signature of a weed corresponds to that of a pre-recorded weed, an “on” signal is transmitted to the spraying unit [150]. Using pulsed microshocks, Bloomer et al. [151] reported that weed control in vegetable and arable crop production can be integrated with cultural controls and inexpensive pre-planting treatments combined with the automatic application of chemical-free weed killers in crops. The experiment conducted by Mathiassen et al. [152] demonstrated that laser treatment of apical meristems led to significant growth reduction and, in some cases, lethal effects on the weeds. Zhu et al. [153] demonstrated that the laser system can accurately detect weeds in corn fields; the robotic arm is capable of precisely aligning the weed, and the blue light laser is effective at removing the weeds. Figure 7 displays an illustration exhibiting the laser-powered weed sensing device.

Reiser et al. [155] created a rotary weeder attachment for an autonomous electric robot. The robot prototype underwent evaluation at a velocity of 0.16 m per second and a depth of operation of 40 mm. Their findings suggested that an automated intra-row weeding robot may serve as a viable alternative to traditional machinery. Chen et al. [156] performed an examination where a photoelectricity detector was placed at the front of the weeding robot. This detector consisted of an array of photocells and a circuit that could receive data from multiple channels. The laser-launching device establishes communication with the robot using a wireless transceiver module. The photoelectric detector determines the location of the light spot via detecting the laser emitted by the laser launching device. Andreasen et al. [157] proposed employing compact autonomous cars outfitted with laser technology as a viable and eco-friendly alternative approach. Laser beams are generated using electrical energy, which can be derived from renewable sources rather than fossil fuels. Deep learning techniques may be employed to precisely detect and distinguish between weeds and agricultural plants. This allows for the accurate focusing and transmission of laser energy through the use of robotic actuators. Due to the precise targeting ability of laser beams, the area that has to be treated for weed control can be significantly decreased compared with conventional weed control methods. As a result, the potential harm to non-target creatures is reduced, and the soil will remain undisturbed in the field, preventing the stimulation of weed seeds to sprout.

However, the weeding power of tiny self-driven vehicles may be restricted, and it is important to exercise caution as the laser beam’s reflections can be hazardous to both humans and animals. Furthermore, the utilization of a laser eradication system is a relatively recent innovation and has not yet been broadly used or commercialized [158]. The use of laser technology for controlling weed seeds may be more suitable for plowed fields in row crops such as sugar beets and maize, as opposed to no-until farming methods. This is because there is a higher danger of burning dry organic matter on the soil surface and perhaps causing a fire [149]. Rakhmatulin et al. [158] designed and evaluated a cost-effective laser-weeding machine prototype. The device was examined on a combination of couch grass (*Elytrigia repens* (L.) *Desv. ex Nevski*) and tomatoes. Three laser types were utilized, with power outputs of 0.3 W, 1 W, and 5 W, respectively. A neural network was programmed to classify the weed plants, while a laser navigation system determined the exact geographic location of a weed. The power needed to harm an herb was contingent upon the plant’s diameter, which was correlated with its length. The laser with a power output of 1 W was inadequate for completely eradicating all weeds and necessitated an excessively lengthy duration of treatment. The 5 W laser exhibited higher efficiency; nevertheless, it additionally caused damage to the crop in the event when the laser beam underwent splitting into two parts during the weeding procedure. There were other obstacles associated with the gadget that require improvement. Specifically, the duration of time exposed must be substantially decreased. In another study by Krupanek et al. [159], the most problematic aspect of automated laser-based weeding equipment functioning is the energy challenge. Contrary to chemical and mechanical methods of weed removal, laser weeding targets only a small portion of the planting area for treatment. Perennial weeds regrow from the belowground parts after the laser destroys the aerial shoots. When the laser consistently destroys fresh shoots, it is feasible that the underground reserves may be depleted. However, this would need plenty of treatment with lasers, resulting in greater electrical power usage [160]. Considering functional efficiency, the existing autonomous laser weeding technologies primarily depend on sequential mechanisms with just one or two controlled axes. However, these mechanisms can hardly fulfill the demands for both high accuracy and flexible action. The precision of placement was assessed through experiments carried out in both laboratory and field circumstances. The mean errors in position at a distance of 535 mm were found to be 0.62 mm. Additionally, the dynamic weeding effectiveness was measured to be approximately 0.72 s/weed, with a dwell time of 0.64 s at a tracking velocity of 0.1 m/s [161]. Using laser-driven sensing for weed detection has been demonstrated to be an innovative method of providing precise and effective weed control while minimizing the need for chemical herbicides. While this technology is expected to have widespread adoption in agricultural practices in the near future, challenges such as energy consumption, targeting accuracy, sensor integration, and environmental sensitivity still need to be addressed prior to its broader adoption.

### 4.2. Seed Mapping

The application of interpolation to calculate weed seedling abundance from geographically provided information has become more common due to the current curiosity in defining the geographical spread of weeds and examining their connection with site features. Furthermore, growers and experts who are embracing aspects of site-specific agricultural strategies are employing interpolation techniques to map weed populations and soil attributes [162]. Real-time kinematic differential GPS (RTK-GPS) may be used to record the locations of seeds during sowing. A seed sensor detects the seeds as they descend from the machine into the ground. Applying this technique, any plants identified in areas other than where the agricultural seed was intentionally sown would be categorized as weeds. Such systems commonly utilize RTK-GPS for accurate place detection and an optical sensor to identify seeds as they are deposited into the soil during planting [163]. Comparing these graphic illustrations of relative weed placements helps anticipate weed spectra and plant biodiversity alterations [164].

**Figure 8:**
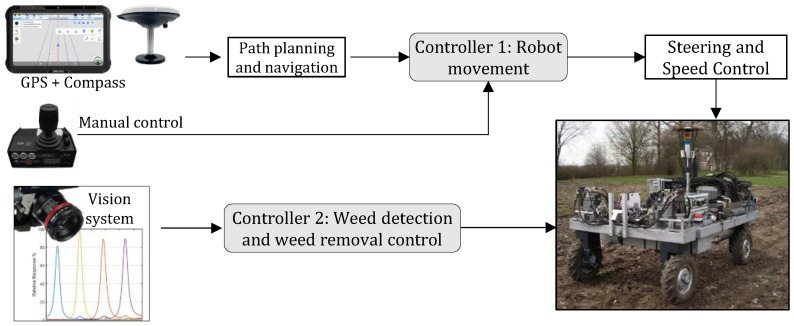
The management system conceptualization for the self-navigating function of the RTK-GPS weeding machine, reprinted from [170,171].

Scientists have examined the precision of automated RTK-GPS agriculture seed mapping systems used in the process of planting. Ehsani et al. [165] point to a seed mapping technique that could promote plant-specific or ultra-precision cultivation. For weed management purposes, it is recommended to employ a precise crop seed map in combination with a plant leaf sensor as a less computationally complicated approach for detecting weeds compared with some machine vision approaches. Experimental testing was carried out to assess the precision of this particular technology for automatically mapping maize seeds in an agricultural area. The seeds were consistently located within a distance of 34 mm from the plant during germination, with a variation between 30 and 38 mm. Griepentrog et al. [166] evaluated a seeding device that utilized optical seed drop sensors and RTK-GPS for location sensing in order to autonomously generate a seed map of a sugar beet field. Average discrepancies ranging from 16 to 43 mm were detected between the GPS seed map created autonomously and the actual plant position after emergence. The specific amount of inaccuracy depended on the speed of the vehicle and the distance between seeds. Potential sources of location errors in the RTK-GPS system were identified as the accuracy of the system itself, the movement of the planter relative to the GPS antennae, the motion of the seed after passing through the optical seed sensors (such as bouncing in the furrow), and soil-related factors (such as clods) that can affect the location of the emerged plant compared to the initial seed location. The optimal results were achieved when the seed was sown at a relative ground speed of zero. Abidine et al. [167] showcased an analogous RTK-GPS system designed to automate the process of pinpointing the location of plants in order to process tomato transplants. An instance of an RTK-GPS robot and its performance are shown in Figure 8.

In their research, Pflanz et al. [168] conducted an experiment to evaluate the performance of an image classifier that utilized a Bag of Visual Words (BoVW) architecture. The classifier was used to map different varieties of weed using a tiny, unmanned aircraft system (UAS) equipped with a commercial camera. The UAS flew at a low elevation during the experiment. The image classifier underwent training using support vector machines, following the creation of a visual dictionary comprising local attributes extracted from a multitude of acquired UAS photos. They utilized a window-based approach to analyze the models and map the presence of weeds in the UAS data. The UAS flying campaign was conducted above a wheat field that was heavily infected with weeds. Photos were captured at altitudes ranging from 1 to 6 m. A total of 25,452 weed plants were identified and labeled at the species level using UAS photos. Additionally, wheat and soil were included as background classes for the purpose of training and validating computational models. The findings demonstrated that the BoVW model achieved a high level of precision in distinguishing individual plants of *Matricaria recutita* L. (88.60%), *Papaver rhoeas* L. (89.08%), *Viola arvensis* M. (87.93%), and winter wheat (94.09%) on the produced maps.

Remote sensing utilizing multispectral aerial imaging can offer precise weed maps, particularly during the advanced weed phenological phases. However, pictures obtained from higher-resolution satellites and UAVs still require analysis. Hyperspectral photographs yield precise maps throughout both early and late phenological phases, either at farm size or on a medium geographical scale. Nevertheless, the present running expenses of this technology are excessively high, making it unprofitable. Precise outcomes can be achieved in investigations of weed seedling recognition on the ground, particularly when conducted on a moderate-sized agricultural operation. The development of a robust and adaptable classifier capable of accurately identifying soil, weeds, and crops in various scenarios continues to be the primary obstacle in this field of science. The primary constraints of distant and proximal sensing may be succinctly characterized as follows: (i) the temporal and educational demands associated with adopting new technological advancements, and (ii) the exorbitant expenses of the technology and the absence of equipment compatibility. Potential solutions could involve (1) establishing a service for advice that offers technical assistance, agronomic expertise, and specialized training programs; (2) creating and enforcing standardized and cost-effective regulations; (3) conducting extensive research on advanced imaging technologies such as high-quality satellite images using object-oriented image analysis and pan-sharpened imagery, as well as UAVs; and (4) facilitating the transformation of existing prototypes of robotic weeding into marketable products [169].

### 4.3. LIDAR and ToF

LIDAR is a method of remote sensing that accurately measures the distance between an instrument and an object of interest [172]. LIDAR sensors generate pulsed light waves that are reflected back to the device after bouncing off nearby objects. The lengths that the pulses travel are determined via calculating the time it takes for all the pulses to return to the generator. Additionally, they are utilized for estimating agricultural biomass, characterizing phenotypes, and monitoring crop growth, among other applications. A LIDAR system and its data may be applied to quantify spray drift and identify soil characteristics [173]. The application of LIDAR has emerged as a highly inventive field in scanning using laser, remote-sensing, and object identification systems in recent years. This technique is likely prominent owing to its ability to precisely identify buildings or zones of interest with millimeter-level accuracy. Additionally, it has the capability to emphasize discrepancies and anomalies, such as the deterioration of surfaces and the proliferation of plants. The subsequent four classifications of modern LIDAR uses are as follows: (1) estimating metrics connected to crops; (2) digitizing trees and plants; (3) developing vision systems for detecting objects and navigating; and (4) providing management and decision-making assistance [174]. In laboratory trials, Weiss et al. [121] achieved an accuracy of about 99% in classifying six plant species employing LIDAR. The researchers used fundamentally derived 3D characteristics, and their experimental setup incorporated the variables encountered in actual field situations, such as the presence of adjacent weeds and plant occlusion. In a study by Shahbazi et al. [175], LIDAR sensors were evaluated for the purposes of detecting and precisely determining the location of weeds. To investigate the detection capabilities of LIDAR, two experiments were conducted using synthetic objects that simulated weeds. These objects varied in length and diameter. The findings demonstrated that the capability to identify the target at various scanning distances from the LIDAR device was directly impacted by the object’s size and its alignment with LIDAR. A third experiment was conducted in a wheat field to assess the ability of a stationary LIDAR device to identify distinct weed species at varying heights above the crop canopy. The findings indicated that the LIDAR technology successfully identified all the weeds in the wheat plot via analyzing the variations in their height. Andújar et al. [176] conducted research to assess the precision and efficiency of a LIDAR sensor in detecting and distinguishing between maize plants, weeds, and soil cover. This was achieved through analyzing distance and reflection data. The research builds upon a prior investigation conducted in a maize field in Spain, where a LIDAR sensor was employed to measure the height pattern as the only indicator. The existing approach uses a blend of the two indices described. The test was conducted in a maize field during growth stages 12–14, at 16 various points chosen to encompass a wide variety of weed densities. The three weed species included in the study were *Echinochloa crus-galli* L., *P. Beauv*., *Lamium purpureum* L., *Galium aparine* L., and *Veronica persica* Poir. A field LIDAR sensor was positioned on a tripod, directed toward the space between rows, with its horizontal axis and field of view oriented vertically downwards toward the ground. It scanned a vertical plane that may have contained plants. Following the collection of LIDAR data, which includes distance and reflection measurements, the heights of the plants were promptly assessed using a suitable approach. To achieve this objective, digital photographs were captured of every selected region. The data revealed a strong connection between the height recorded via LIDAR and the true heights of the plants (R^2^ = 0.75). The precision of the sensor was validated through conducting a binary logistic regression analysis between the presence or absence of weeds and the sensor readings, namely the LIDAR height and reflection values. This enabled the differentiation of plants from the ground with a precision of up to 95%. Furthermore, a canonical discrimination analysis (CDA) successfully distinguished mainly between soil and vegetation and, to a lesser degree, between crops and weeds. The results obtained by Andújar et al. [176] demonstrated that LIDAR sensors show great potential for detecting and distinguishing weeds. They offer notable benefits compared with other non-contact ranging instruments, including a higher sampling resolution and the capability to scan at high sampling rates. However, the industrial application of these sophisticated innovations appears to be quite limited [177]. It is important to note that LIDAR is limited due to its inability to distinguish between weeds and crops of similar heights and structures, particularly in dense vegetation or complex terrain, and its performance can be affected by atmospheric conditions such as dust, fog, or rain. Moreso, LIDAR data processing requires a high level of computational power, particularly for real-time applications. Using LIDAR in conjunction with other sensors, such as vision systems, entails calibration and data interpretation challenges, as well as being expensive and energy-intensive. Therefore, it is not a practical tool for widespread use in agriculture.

Recently, time-of-flight (ToF) cameras have garnered interest due to their capability to produce a depth picture at high speeds of video frames. ToF cameras are well-suited for real-time 3D activities, such as tracking or object position determination [178]. Computer vision is especially beneficial for robots working in areas with tall plants like maize (*Zea mays*) and sorghum (*Sorghum bicolor*), where GPS signals can sometimes not be accessible. Nonetheless, the advancement of under-canopy navigational devices remains a subject of ongoing study. Gai et al. [179] created a system that uses a ToF camera to navigate beneath a canopy based on visual information. As active 3D image sensors, they are more resistant to the effects of ambient sunlight compared with passive detection systems. While the existing 3D image processing algorithms for plant recognition can offer quite accurate and detailed 3D spatial data, they are still considered rudimentary, according to the literature. A maize plant spacing system utilizing a ToF camera was created in a study by Nakarmi and Tang [180]. The system successfully obtained a recognition rate of 98% for corn plants, specifically targeting crop plants that were noticeably higher than weeds or other items. However, this method was not designed and evaluated for situations in which crop plants and weeds had similar heights. Furthermore, the rapid processing rate of the equipment was insufficient to fulfill the speed criteria of an automated weeding system. As a result, they were merely used to distinguish between areas with and without vegetation [176]. As a result of signal interference and the variable reflectivity of plant surfaces, ToF sensors often have difficulty measuring distances accurately and distinguishing between plants that are close to each other. Additionally, the quality of depth data may be compromised due to environmental factors such as ambient light, rain, fog, and dust. Due to their limited range, they frequently struggle to detect small weeds close to the ground, especially when these weeds have similar heights to the crops. Additionally, in order to integrate ToF sensors into agricultural robots, it is necessary to address issues such as sensor noise and data interpretation issues.

### 4.4. Ultrasonic Systems

Ultrasonic sensors utilize sound waves with frequencies over 20 kHz to recognize items that are nearby. In the industrial sector, ultrasonic detectors are employed in robotics as well as various projects where accurate detection of existence, closeness, or location is necessary [181]. Andújar et al. [182] utilized an ultrasonic proximity device to identify weeds via measuring crop height. It was postulated that the areas infested with weeds contain a greater quantity of biomass compared with non-infested regions, and this may be ascertained via measures of plant height. Discriminant evaluation was applied to assess the potential for discriminating against weed groups. The ultrasonic measurements accurately distinguished between regions infested with weeds and non-infested regions, with a success rate of up to 92.8%. Andújar et al. [31] proposed a novel method for automatically distinguishing between grasses and broad-leaved weeds via utilizing their respective heights. A tractor was equipped with an ultrasonic sensor that was positioned on the front of the vehicle. The sensor was directed downward, perpendicular to the ground, in the space between the rows of crops. A monitoring system was used to accurately determine the location of the sensor and record the echoes that were reflected by the ground or the different layers of leaves. Observations were collected at several places with varying concentrations of grasses (*Sorghum halepense*) and broad-leaved weeds (*Xanthium strumarium* and *Datura* spp.). The sensor outputs enabled the differentiation of homogeneous grass fields (with a success rate of up to 81%) and homogeneous broad-leaved weed fields (with a success rate of up to 99%). Chandra Swain et al. [183] constructed advanced and cost-effective ultrasonic measurement equipment specifically designed to identify and locate weeds and bare patches in wild blueberry farms. The specifically developed farm-motorized vehicle had ultrasonic sensors installed alongside its back wheels. The Trimble Ag GPS 332 was positioned above the sensors to accurately determine the precise coordinates of the sensor data points for mapping purposes. The linear regression analysis revealed a strong correlation between the actual heights and the sensor heights, with an R-squared value of 0.98. The survey of the field to detect weeds and bare areas was conducted at a velocity of 0.54 m/s. As reported by Amid et al. [184], the ultrasonic robot shows promise as a dependable and automated method for weed management in greenhouse cucumber production, with the possibility for commercialization. Rohani [185] performed research using a well-equipped setup capable of transmitting and receiving ultrasonic waves at a frequency of 40 kHz. The study successfully recognized five weed species: Portulacacea, *Chenopodium album* L., *Tribulus terrestris* L., *Amaranthus retroflexus* L., and *Salsola iberica*. Ultrasonic waves at a frequency of 40 kHz were sent toward the weed canopy and then detected by an ultrasonic receiver. These signals were subsequently sent to a laptop and saved in MATLAB 2013. The software utilized an artificial neural network (ANN) to differentiate between different types of weeds and finally classify them. In summary, the findings indicated that by removing around 20% of the ineffective signal characteristics, ANN may achieve a maximum detection accuracy of up to 80%. Nevertheless, these advanced sensor systems have not yet been widely used due to their limited accessibility for farmers and the additional efforts required for their use and administration [182]. Similarly to other sensors discussed previously, ultrasonic sensors are highly sensitive to environmental conditions, such as wind, rain, and soil moisture, which can disrupt sound wave propagation and cause inaccurate measurements. In addition, due to the low resolution of ultrasonic sensors, it is often difficult to accurately identify individual weed species, particularly in dense vegetation or fields with different heights of plants. Background noise can interfere with them, causing false readings and reducing the reliability of weed detection, and the range of the sensors limits their effectiveness in detecting weeds that are either near or far from them.

## 5. Field Applications and Technical Challenges

The implementation of robotic weeding faces several technological obstacles and constraints related to the technologies [186]. These issues are crucial to overcome in order to further improve and enhance the effectiveness of computerized weed management systems. An important obstacle is the durability and dependability of sensing technology used for identifying and distinguishing weeds in agricultural settings [138]. Conventional RGB cameras, although commonly used, generally face challenges with distinguishing small variations between crops and weeds, especially in complicated, disorganized situations with various conditions of light and levels of foliage. The heterogeneity of soil and crops is a significant difficulty. Variations in soil color, texture, and moisture levels might impact the reflectance characteristics detected by optical equipment, potentially resulting in an incorrect categorization of crops [187]. In particular, in some spectral bands, detectors may struggle to differentiate between dark, damp soil and green weeds due to their similar appearance. Likewise, both crops and weeds can display notable variations in their physical characteristics, such as differences in the form, size, and growth patterns of their leaves. The presence of a high concentration of weeds and their various phases of development provides additional challenges for sensing technologies. Identifying early-stage weeds can be especially difficult since they are tiny and look similar to immature crop plants, making them more important to manage [188]. Substantial blooms of weeds can present challenges because the overlapping foliage and stems can obstruct the identification and targeting of individual plants by cameras [189]. Utilizing self-calibrating sensors that automatically adapt settings based on environmental factors such as illumination, dust, high density of weeds, and humidity may effectively ensure constant function. Adaptive skills are critical for guaranteeing the resilience of robots in different and fluctuating agricultural environments [190]. These issues require the use of high-resolution sensors and advanced data processing skills to precisely recognize and categorize plants at different stages of development and density. In order to address the limits of individual sensors, combining the capabilities of several sensing technologies, such as optical imaging devices, LIDAR, ultrasonic detectors, and thermal cameras, becomes an imperative approach [191]. Every kind of sensor has distinct benefits, for example, optical cameras present highly detailed images, while LIDAR provides accurate spatial readings. By utilizing advanced algorithms, the integration of data collected from diverse sensors may greatly improve the precision and dependability of weed identification. It is important to develop strong sensor fusion algorithms that can integrate and interpret information immediately. The algorithms need to efficiently combine several data streams, providing a complete and accurate depiction of the field that can distinguish minute distinctions between crops and weeds. Precise positioning and movement are essential for the efficient functioning of robotic weeders [139]. Creating sophisticated navigational equipment that uses GPS, inertial measurement units (IMUs), and real-time kinematic (RTK) positioning may improve the accuracy of robotic mobility on farms [192]. Precise navigation guarantees efficient pinpointing of weeds and limits the potential for crop harm. Moreover, it is vital to improve the ability to recognize and avoid obstacles via employing LIDAR and ultrasonic sensors in order to navigate intricate field conditions. These enhancements may aid in the preservation of operating efficiency and the prevention of disruptions triggered by impediments.

Additionally, the need to interpret vast amounts of high-quality visual information in real-time is a major challenge in implementing sensor-based robotic weeding systems [193]. To quickly and accurately identify weeds, it is necessary to use analytically complex algorithms, such as AI and computer vision methods [194]. These algorithms help in making timely decisions and carrying out necessary actions. Cloud-based technological platforms offer the required computational capacity to handle intricate computations involving data, enabling the creation and improvement of advanced models [195]. Moreover, solutions that use cloud computing facilitate the exchange of data and models between various robotic devices and farms, promoting ongoing enhancement and development [196]. Striking a balance between the computational load and the requirement for quick response rates is a crucial technological obstacle. This issue necessitates creative strategies in methodology improvement and hardware architecture in order to attain acceptable results within operational limitations. Furthermore, the robustness and resilience of sensing equipment in agricultural environments, which are marked by elements like particles, dirt, and humidity, provide actual barriers that need to be overcome to guarantee the sustained dependability and effectiveness of robots. Delicate or vulnerable instruments are prone to physical harm and deterioration from the environment, which can compromise the effectiveness and longevity of the system [197]. Hence, it is crucial to have strong and durable packaging for sensors, together with safe structures and sealing initiatives, in order to minimize the negative impact of severe farming circumstances on sensing electronic devices. This will guarantee that the system remains operational and reliable for an extended period of time.

One further technological challenge is ensuring that autonomous weeders will operate harmoniously with the current agricultural machinery and methods. It is crucial to ensure that these new technologies can be seamlessly incorporated into existing farming practices without necessitating significant alterations or interruptions in order to promote their widespread use. The compatibility also applies to the applications and information platforms utilized by growers, requiring them to have the ability to process the data produced by robotic weeders and seamlessly incorporate them with other farm-related instruments. The problem of scalability and flexibility is a substantial engineering obstacle. The sensing system used for robots demands the ability to be simply adjusted to varied land sizes and be versatile enough to operate successfully with diverse crop varieties and agricultural methods [198]. The necessity for adaptability requires structures that are modular and flexible, allowing them to be adapted to various agricultural requirements. Ensuring the adaptability of sensing systems to multiple crops, development phases, and climatic circumstances while maintaining high levels of precision and effectiveness is a multifaceted challenge. Effective collaboration with agricultural scientists and producers may greatly improve the commercial usefulness of sensing technologies in automated weeding [199]. Integrating user input into the engineering workflow guarantees that the technology aligns with the actual demands and preferences of customers. Performing comprehensive field experiments in diverse settings and with varied crops yields useful data for improving sensing technology [200]. These tests enable the identification of practical difficulties and opportunities for upgrading, resulting in the development of systems that are more convenient to use and are efficient. The incorporation of sustainable practices is a fundamental factor in the ongoing creation of autonomous weeding systems. Enhancing the energy productivity of instruments and computational processors has the potential to prolong the operating duration of robotic weeders, hence increasing their viability for extended periods of usage. By using low-power devices and improving systems to maximize energy efficiency, it is possible to decrease total power usage. Applying energy from renewable resources, such as photovoltaic cells, may improve the environmental sustainability of robots and decrease their impact on the ecosystem.

An important barrier to adopting robotic weeding technologies is the lack of qualified professionals with both agricultural knowledge and the technical expertise needed to design, implement, and maintain these systems. Weeding using robots requires a multidisciplinary approach that brings together knowledge from a wide range of fields, including agronomy, robotics, computer science, and engineering. There are, however, a limited number of professionals with the necessary skills to meet the demand for the intersection of these disciplines. An understanding of crop and weed biology, soil conditions, and agricultural practices is required in order to design and fine-tune robotic weeders. Additionally, expertise in robotics, machine learning, and sensor technologies is essential for developing effective weed detection algorithms and integrating them into autonomous systems. This has resulted in farmers and agricultural businesses having difficulty finding qualified technicians capable of operating and maintaining robotic weeders, troubleshooting issues, and optimizing the system’s performance. Consequently, farmers may be unwilling to adopt these technologies, increasing downtime and reducing efficiency. Table 4 provides a concise overview of the constraints and suggested solutions in the field of robotic sensing.

In order to improve the accuracy and reliability of weed detection systems, sensor fusion is a critical solution. It involves combining data from multiple sensors. A combination of LIDAR and RGB or multispectral cameras, for instance, would be capable of overcoming the limitations of each sensor individually. In spite of its high ability to map the 3D structure of the environment, LIDAR is susceptible to problems when faced with reflective surfaces and poor weather conditions. It has been demonstrated that by incorporating RGB or multispectral data, which provide detailed color and spectral information, the system is capable of compensating for the weaknesses of LIDAR, resulting in more robust weed detection even in challenging conditions [209] and interpreting the combined data using machine learning algorithms. As a result of this approach, weed detection accuracy is enhanced, and the system is able to perform effectively in a wider range of environmental conditions.

To improve the processing of complex sensor data, it is necessary to implement advanced algorithms, particularly those involving machine learning and artificial intelligence. When hyperspectral imaging is integrated with artificial intelligence, for instance, the computational burden can be reduced via focusing on the most informative spectral bands instead of processing the entire spectrum [73]. In addition to improving the efficiency of the system, this also reduces the cost and complexity associated with the processing of hyperspectral data. Moreover, algorithms that adapt to changing environmental conditions, such as varying light intensity or shadowing, can enhance the performance of RGB and multispectral sensors. A number of techniques, including adaptive exposure control, image enhancement, and the use of convolutional neural networks (CNNs), may enhance the robustness of these sensors in field applications. In addition, sensors should be deployed and calibrated according to the environmental conditions in which they will be used. The FLIR E5 thermal camera, for example, can be adversely affected by extreme weather conditions and temperature variations. Combining thermal imaging with other sensing modalities, such as NIR or visual cameras, can mitigate these disadvantages via providing complementary data that can compensate for the limitations of thermal sensors [210]. It is also possible to improve the accuracy of weed detection through implementing thermal correction algorithms, which help adjust the readings based on known environmental variables. The performance of LIDAR sensors can be significantly improved via protecting them from dust, fog, and reflective surfaces, as well as using filters or algorithms that reduce noise in the process. Moreover, when combining LIDAR with multispectral imaging, different types of vegetation can be differentiated, even when adverse weather conditions prevail [211]. Hardware enhancements are crucial to overcoming sensor limitations. The Intel RealSense D435 camera, for example, can improve the resolution and range of RGB-D cameras, which can be beneficial in addressing issues related to depth perception and low-resolution data. As well as reducing latency and improving real-time performance, it is also possible to upgrade the sensor’s processing unit to handle higher data loads or incorporate edge computing devices to process data locally [212].

For future development directions, advances in AI and machine learning will play a key role in improving weed detection accuracy. Enhanced deep learning models could allow systems to learn more subtle differences between crops and weeds. Additionally, there is room for collaborative robot systems (multirobot swarms) to increase efficiency, where a fleet of smaller robots works together autonomously to cover larger areas more quickly. Integration of sensors and data sharing between robots can enhance performance, but this also requires better communication protocols and data management solutions.

As a final point, there are several approaches that can be taken in order to address the issue of professional scarcity and lack of subject knowledge in robotic weeding: (1) Developing specialized degree programs and certifications that focus on the intersection of agriculture, robotics, and artificial intelligence can help bridge the skills gap. Partnerships between universities, research institutions, and industry can provide students with practical experience in agricultural environments. (2) The sharing of knowledge and resources among technology companies, agricultural organizations, and academic institutions can be enhanced through collaboration. It is also possible to develop training programs tailored to the needs of the industry through this collaboration. (3) Offering continuing education programs to current agricultural and engineering professionals may assist them in acquiring the skills required to operate robotic weeders.

## 6. Conclusions

The increasing trend toward sustainable agriculture has led to innovations in robotic weeding tools that eliminate weeds without chemicals, with an emphasis on methods for improving accuracy, minimizing ecological damage, and reducing operational costs. Some of the sensing and perception solutions, including RGB cameras, LIDAR, multispectral, hyperspectral, and thermal imaging sensors, along with their advantages and drawbacks, were presented and discussed in this study. It can be concluded that the effectiveness of imaging solutions depends on external factors like illumination, shadows, intense sunshine, and low light, which may fluctuate substantially and cause these sensing devices to overlook or falsely identify weeds. For distinguishing crops and weeds based on appearance, plant structure, and height, LIDAR sensors have been employed. However, laser signals can be distorted due to dust, fog, and shiny surfaces like wet leaves, making detection difficult. In addition, their high costs limit their use in cost-sensitive agricultural applications. RGB cameras are often enhanced via using multispectral and hyperspectral imaging sensors, which generate vast amounts of data and can recognize small changes in plant surface reflectance, enabling spectral signature-based weed species detection. However, interpreting this information requires calibration, complex calculations, and significant computing capacity to analyze in real time, which is expensive and time-consuming, making it challenging to use in robotic systems. Thermal detectors can be useful for capturing plant temperature, which is correlated to water content and the rate of metabolism. However, they are susceptible to external factors like ambient temperature and wind, which may alter plant thermal patterns and cause false findings. Additionally, thermal images have lower resolution than RGB or multispectral images, making it hard to distinguish small or closely placed weeds. Our study showed that research and development toward building a commercial robotic weeding system is leaning more toward sensor integration and solutions that benefit from machine learning, AI, and sophisticated algorithms that interpret complex sensor data to address existing limitations and provide real-time weed identification and decision-making. Such robots can also learn from prior events to improve detection while decreasing inaccuracies. While robotic weeding innovations have progressed significantly in the last 20 years, our study concludes that sensing reliability, data analysis, and ecological adaptability remain issues. It is expected that robotic weeders will eventually substitute chemical-based weed management in sustainable agriculture as technological innovations mature. Future studies may involve investigation of the energy efficiency and battery life of robotic weeding systems, especially those that often need to cover large fields and prolonged operational times can drain batteries quickly, reducing productivity. There is a strong need for further developments in energy management, such as optimizing power consumption and exploring alternative energy sources like solar power.

## Figures and Tables

**Table 2:** Several commercial robotic weeders and their features.

Robot	Manufacturer	Crop	Features
See & Spray [41]	Blue River Technology	Soybeans, corn	Reduces herbicide application, precise weed targeting
EcoRobotix [42]	EcoRobotix	Sugar beets, vegetables	Solar-powered, reduces chemical use, eco-friendly
Dino [43]	Naïo Technologies	Carrots, lettuce	Autonomous navigation, reduces manual labor, precise weeding
Titan [44]	FarmWise	Lettuce, spinach	AI-driven, eliminates need for herbicides, sustainable
Robocrop [45]	Garford	Sugar beets, lettuce	High-resolution cameras, precise mechanical weeding
Avo [46]	Ecorobotix	Soybeans, corn	Multispectral cameras, AI for precise targeting
Ladybird [47]	University of Sydney	Spinach, lettuce	Machine learning for weed identification, autonomous weeding

**Table 3:** Comparison of spectral, hyperspectral, multispectral, and NIR-Hyperspectral sensing methods. Sources: Tsoulias et al. [127], Che’Ya et al. [128], and de Castro et al. [129].

Feature	Spectral Sensing	Multispectral	Hyperspectral	NIR-Hyperspectral
Number of bands	Typically 1–3 bands (e.g., NIR or specific wavelength)	3–10 bands	Hundreds of narrow bands across the electromagnetic spectrum	Hundreds of bands, focused on the NIR region
Data volume	Low	Moderate	High	High
Cost	Low	Moderate	High	High
Complexity	Low	Moderate	High	High
Accuracy	Moderate, limited to specific conditions	Higher than spectral, good for differentiating some weed species	Very high, capable of distinguishing even similar species	High; particularly effective in vegetation analysis
Real-time processing	Suitable for real-time processing due to lower data volume	Possible but requires good computational resources	Challenging due to large data volume, typically requires offline processing	Challenging but more focused, reducing data volume slightly
Sensitivity to environment	High (affected by light, soil moisture, etc.)	Moderate, but still sensitive to environmental factors	Less sensitive, but still affected by light and other conditions	Lower sensitivity, optimized for NIR reflectance in vegetation
Field of application	Basic weed detection in controlled environments	Precision agriculture, broad-acre weed mapping, UAV applications	Advanced research, detailed weed stress analysis, species identification	Detection of moisture content, plant health, specific weed identification
Integration with robotics	Easy to integrate, especially in low-cost systems	Often integrated with UAVs and ground robots for field mapping	Difficult to integrate due to complexity, used in high-end systems	Complex, but highly valuable in agricultural robotics
Challenges	Environmental variability, limited spectral information	Calibration issues, overlapping spectral signatures	High cost, data overload, complex data analysis required	High cost, requires advanced algorithms, real-time processing

**Table 4:** Limitations of sensors installed on robotic weeders.

Weeding Platform	Sensor Type, Model	Sensor Limitations
AgBot II [201]	LiDAR, SICK LMS111	Affected by dust, fog, and reflective surfaces
Naio Dino [202]	RGB, Basler ace acA2040-90uc	Sensitive to lighting conditions and shadows
Rowbot [203]	Multispectral, MicaSense RedEdge-MX	Expensive and complex data processing required
Blue River See & Spray [204]	RGB, Canon EOS 70D	Limited spectral range and sensitivity to light conditions
EcoRobotix [205]	Hyperspectral, Headwall Photonics Nano-Hyperspec	High cost and requires complex data interpretation
Ecorobotix AVO [206]	Multispectral, Sentera Quad	Expensive and requires significant computational resources
Robovator [11]	NIR, Sentek Dynamics M7	Limited by environmental conditions such as lighting
F. Poulsen Engineering Robovator	LiDAR, Velodyne VLP-16	Can be expensive and affected by weather conditions
Bosch Deepfield Robotics BoniRob [207]	RGB-D, Intel RealSense D435	Limited range and resolution; affected by lighting
Weedmaster [208]	Thermal Camera, FLIR E5	Less effective in extreme temperature variations and weather conditions

## Data Availability

Data are contained within the article.

## References

[1] Fontanelli M., Frasconi C., Martelloni L., Pirchio M., Raffaelli M., Peruzzi A. (2015). Innovative strategies and machines for physical weed control in organic and integrated vegetable crops. Chem. Eng. Trans..

[2] Steward B., Gai J., Tang L. (2019). The use of agricultural robots in weed management and control. The Use of Agricultural Robots in Weed Management and Control.

[3] Saunders J.T., Greer G., Bourdôt G., Saunders C., James T., Rolando C., Monge J., Watt M.S. (2017). The economic costs of weeds on productive land in New Zealand. Int. J. Agric. Sustain..

[4] Gharde Y., Singh P., Dubey R., Gupta P. (2018). Assessment of yield and economic losses in agriculture due to weeds in India. Crop. Prot..

[5] WSSA (2016). WSSA Calculates Billions in Potential Economic Losses from Uncontrolled Weeds. https://wssa.net/2016/05/wssa-calculates-billions-in-potential-economic-losses-from-uncontrolled-weeds/.

[6] Axbom S., Ralsgård L. (2018). Design of an Autonomous Weeding Vehicle Used in the Agricultural Industry. Master’s Thesis.

[7] Rad A.K., Astaikina A., Streletskii R., Zarei M., Etesami H., Bandh S.A., Shafi S. (2022). Chapter 10—Fungicide and pesticide fallout on aquatic fungi. Freshwater Mycology.

[8] Bakker T., Asselt K., Bontsema J., Müller J., van Straten G. (2010). Systematic design of an autonomous platform for robotic weeding. J. Terramechanics.

[9] Shamshiri R.R., Balasundram S.K., Rad A.K., Sultan M., Hameed I.A. (2022). An Overview of Soil Moisture and Salinity Sensors for Digital Agriculture Applications.

[10] Shamshiri R.R., Sturm B., Weltzien C., Fulton J., Khosla R., Schirrmann M., Raut S., Basavegowda D.H., Yamin M., Hameed I.A. (2024). Digitalization of agriculture for sustainable crop production: A use-case review. Front. Environ. Sci..

[11] Fennimore S.A., Cutulle M. (2019). Robotic weeders can improve weed control options for specialty crops. Pest Manag. Sci..

[12] Su W.-H. (2020). Crop plant signaling for real-time plant identification in smart farm: A systematic review and new concept in artificial intelligence for automated weed control. Artif. Intell. Agric..

[13] Bajwa A.A., Mahajan G., Chauhan B.S. (2015). Nonconventional weed management strategies for modern agriculture. Weed Sci..

[14] Pérez-Ruíz M., Slaughter D.C., Fathallah F.A., Gliever C.J., Miller B.J. (2014). Co-robotic intra-row weed control system. Biosyst. Eng..

[15] Wang A., Zhang W., Wei X. (2019). A review on weed detection using ground-based machine vision and image processing techniques. Comput. Electron. Agric..

[16] Dyrmann M., Karstoft H., Midtiby H.S. (2016). Plant species classification using deep convolutional neural network. Biosyst. Eng..

[17] Kanimozhi T., Umarani S. (2023). A comparative study of energy-efficient clustering protocols for WSN-internet-of-things. Int. J. Hydromechatronics.

[18] Ayua S.I. (2023). Random Forest Ensemble Machine Learning Model for Early Detection and Prediction of Weight Category. J. Data Sci. Intell. Syst..

[19] Khalil Z.H., Khaleel A.H. (2023). Design of a Multilayer Perceptron Network Based on the Normalized Histogram to Develop Yields Predictive Model. J. Data Sci. Intell. Syst..

[20] Chinthamu N., Karukuri M. (2023). Data Science and Applications. J. Data Sci. Intell. Syst..

[21] Ward S.M., Webster T.M., Steckel L.E. (2013). Palmer Amaranth (Amaranthus palmeri): A Review. Weed Technol..

[22] Holm L., Doll J., Holm E., Pancho J.V., Herberger J.P. (1997). World Weeds: Natural Histories and Distribution.

[23] Parker C., Fryer J. (1975). Weed Control Problems Causing Major Reductions in World Food Supplies.

[24] Holm L.G., Plucknett D.L., Pancho J.V., Herberger J.P. (1977). The World’s Worst Weeds. Distribution and Biology.

[25] Chauhan B.S., Johnson D.E. (2011). Ecological studies on Echinochloa crus-galli and the implications for weed management in direct-seeded rice. Crop. Prot..

[26] Warwick S.I., Black L.D. (1983). THE BIOLOGY OF CANADIAN WEEDS: 61. *Sorghum halepense* (L.) PERS. Can. J. Plant Sci..

[27] Dekker J. (2003). The foxtail (Setaria) species-group. Weed Sci..

[28] VGN Weeding Robots Put Farms in Better Control. https://vegetablegrowersnews.com/article/weeding-robots-put-farms-in-better-control/.

[29] Pearce R. The Robots Are Here, and Ready to Weed Your Field. Country-Guide. https://www.country-guide.ca/machinery/the-robots-are-here-and-ready-to-weed-your-field/.

[30] Pérez A.J., López F., Benlloch J.V., Christensen S. (2000). Colour and shape analysis techniques for weed detection in cereal fields. Comput. Electron. Agric..

[31] Andújar D., Escolà A., Dorado J., Fernández-Quintanilla C. (2011). Weed discrimination using ultrasonic sensors. Weed Res..

[32] Mulla D.J. (2013). Twenty five years of remote sensing in precision agriculture: Key advances and remaining knowledge gaps. Biosyst. Eng..

[33] Gongal A., Amatya S., Karkee M., Zhang Q., Lewis K. (2015). Sensors and systems for fruit detection and localization: A review. Comput. Electron. Agric..

[34] Hamuda E., Mc Ginley B., Glavin M., Jones E. (2017). Automatic crop detection under field conditions using the HSV colour space and morphological operations. Comput. Electron. Agric..

[35] Mahlein A.-K. (2015). Plant Disease Detection by Imaging Sensors—Parallels and Specific Demands for Precision Agriculture and Plant Phenotyping. Plant Dis..

[36] Belbachir A.N., Göbel P.M., Belbachir A.N. (2010). Smart Cameras: A Historical Evolution. Smart Cameras.

[37] Zhang D., Wei B. (2017). Robotics and Mechatronics for Agriculture.

[38] Linton J. (2009). SRDC Grower Group Innovation Project Final Report Precision Spot Spraying System: It Works in Grains Will It Work in Cane?.

[39] Riczu P., Tamás J. (2013). Applicability of precision weed detection technologies. Acta Agrar. Debreceniensis.

[40] Kool J., de Jonge E., Nieuwenhuizen A., Braam H. (2023). Green on Green Weed Detection: Finding Weeds in a Soybean Crop in Brazilian Fields with the Rometron WEED-IT Sensor: Intermediary Report.

[41] Deere SEE & SPRAY™ ULTIMATE Targeted, In-Crop Spraying. https://www.deere.com/en/sprayers/see-spray-ultimate/.

[42] Ghaly A., Ibrahim M. (2022). Mechanization of Weed Management in Sugar Beet. Sugar Beet Cultivation, Management and Processing.

[43] Naio-Technologies DINO: The World’s First Row-Straddling Weeding Robot to Reach The market. https://www.naio-technologies.com/en/news/dino-the-worlds-first-row-straddling-weeding-robot-to-reach-the-market/.

[44] The Robot Report Farm Wise Delivers Cultivation as a Service for Farmers. https://www.therobotreport.com/farmwise-delivers-cultivation-as-a-service-for-farmers/.

[45] Collings A. (2006). Robocrop Helps with Weed Removal. https://www.fwi.co.uk/arable/robocrop-helps-with-weed-removal.

[46] Ecorobotix AVO. https://ecorobotix.com/en/avo/.

[47] Robohub Ladybird with James Underwood. https://robohub.org/robots-ladybird/.

[48] Romeo J., Guerrero J.M., Montalvo M., Emmi L., Guijarro M., Gonzalez-de-Santos P., Pajares G. (2013). Camera sensor arrangement for crop/weed detection accuracy in agronomic images. Sensors.

[49] Ceccarelli A., Secci F. (2022). RGB Cameras Failures and Their Effects in Autonomous Driving Applications. IEEE Trans. Dependable Secur. Comput..

[50] NEG Image Sensor Cover Glass. https://www.neg.co.jp/en/rd/topics/product-cover-glass/.

[51] Saraswathi S., Eduardo A., Ricardo M., André V., Pedro D.G. (2020). Automated Weed Detection Systems: A Review. KnE Eng..

[52] Juwono F.H., Wong W.K., Verma S., Shekhawat N., Lease B.A., Apriono C. (2023). Machine learning for weed–plant discrimination in agriculture 5.0: An in-depth review. Artif. Intell. Agric..

[53] Gerhards R., Andújar Sanchez D., Hamouz P., Peteinatos G.G., Christensen S., Fernandez-Quintanilla C. (2022). Advances in site-specific weed management in agriculture—A review. Weed Res..

[54] Burkart A., Hecht V.L., Kraska T., Rascher U. (2018). Phenological analysis of unmanned aerial vehicle based time series of barley imagery with high temporal resolution. Precis. Agric..

[55] Chawade A., van Ham J., Blomquist H., Bagge O., Alexandersson E., Ortiz R. (2019). High-Throughput Field-Phenotyping Tools for Plant Breeding and Precision Agriculture. Agronomy.

[56] Lu Y., Young S. (2020). A survey of public datasets for computer vision tasks in precision agriculture. Comput. Electron. Agric..

[57] Sudars K., Jasko J., Namatevs I., Ozola L., Badaukis N. (2020). Dataset of annotated food crops and weed images for robotic computer vision control. Data Brief.

[58] Gené-Mola J., Vilaplana V., Rosell-Polo J.R., Morros J.-R., Ruiz-Hidalgo J., Gregorio E. (2019). Multi-modal deep learning for Fuji apple detection using RGB-D cameras and their radiometric capabilities. Comput. Electron. Agric..

[59] Nguyen T.T., Vandevoorde K., Wouters N., Kayacan E., De Baerdemaeker J.G., Saeys W. (2016). Detection of red and bicoloured apples on tree with an RGB-D camera. Biosyst. Eng..

[60] Huang A., Bachrach A., Henry P., Krainin M., Maturana D., Fox D., Roy N. (2011). Visual Odometry and Mapping for Autonomous Flight Using an RGB-D Camera. https://www.researchgate.net/publication/228941748_Visual_Odometry_and_Mapping_for_Autonomous_Flight_Using_an_RGB-D_Camera.

[61] Putra B.T.W., Soni P. (2020). Improving nitrogen assessment with an RGB camera across uncertain natural light from above-canopy measurements. Precis. Agric..

[62] Costa L., Nunes L., Ampatzidis Y. (2020). A new visible band index (vNDVI) for estimating NDVI values on RGB images utilizing genetic algorithms. Comput. Electron. Agric..

[63] Gracia-Romero A., Vergara-Díaz O., Thierfelder C., Cairns J.E., Kefauver S.C., Araus J.L. (2018). Phenotyping Conservation Agriculture Management Effects on Ground and Aerial Remote Sensing Assessments of Maize Hybrids Performance in Zimbabwe. Remote Sens..

[64] Matese A., Di Gennaro S.F. (2018). Practical Applications of a Multisensor UAV Platform Based on Multispectral, Thermal and RGB High Resolution Images in Precision Viticulture. Agriculture.

[65] de Lima D.C., Saqui D., Ataky S., Jorge L.A.d.C., Ferreira E.J., Saito J.H., Rodrigues J.M.F., Cardoso P.J.S., Monteiro J., Lam R., Krzhizhanovskaya V.V., Lees M.H., Dongarra J.J., Sloot P.M.A. (2019). Estimating Agriculture NIR Images from Aerial RGB Data. Proceedings of the Computational Science—ICCS.

[66] Endres F., Hess J., Sturm J., Cremers D., Burgard W. (2014). 3-D Mapping With an RGB-D Camera. IEEE Trans. Robot..

[67] Marin J.F., Mostaza-Colado D., Parra L., Yousfi S., Mauri P.V., Lloret J. Comparison of performance in weed detection with aerial RGB and thermal images gathered at different height. Proceedings of the ICNS 2021.

[68] Gašparović M., Zrinjski M., Barković Đ., Radočaj D. (2020). An automatic method for weed mapping in oat fields based on UAV imagery. Comput. Electron. Agric..

[69] Rasmussen J., Nielsen J., Streibig J.C., Jensen J.E., Pedersen K.S., Olsen S.I. (2019). Pre-harvest weed mapping of Cirsium arvense in wheat and barley with off-the-shelf UAVs. Precis. Agric..

[70] Hassanein M., El-Sheimy N. (2018). An Efficient Weed Detection Procedure Using Low-Cost Uav Imagery System for Precision Agriculture Applications. Int. Arch. Photogramm. Remote Sens. Spat. Inf. Sci..

[71] Fu L., Gao F., Wu J., Li R., Karkee M., Zhang Q. (2020). Application of consumer RGB-D cameras for fruit detection and localization in field: A critical review. Comput. Electron. Agric..

[72] García-Berná J.A., Ouhbi S., Benmouna B., García-Mateos G., Fernández-Alemán J.L., Molina-Martínez J.M. (2020). Systematic Mapping Study on Remote Sensing in Agriculture. Appl. Sci..

[73] Slaughter D.C., Giles D.K., Downey D. (2008). Autonomous robotic weed control systems: A review. Comput. Electron. Agric..

[74] Lottes P., Behley J., Milioto A., Stachniss C. (2018). Fully Convolutional Networks With Sequential Information for Robust Crop and Weed Detection in Precision Farming. IEEE Robot. Autom. Lett..

[75] Hashemi-Beni L., Gebrehiwot A., Karimoddini A., Shahbazi A., Dorbu F. (2022). Deep Convolutional Neural Networks for Weeds and Crops Discrimination From UAS Imagery. Front. Remote Sens..

[76] Rai N., Zhang Y., Ram B., Schumacher L., Kiran R., Bajwa S., Sun X. (2023). Applications of deep learning in precision weed management: A review. Comput. Electron. Agric..

[77] Verhoeven G. (2008). Imaging the invisible using modified digital still cameras for straightforward and low-cost archaeological near-infrared photography. J. Archaeol. Sci..

[78] Jung C., Zhou K., Feng J. (2020). Fusionnet: Multispectral Fusion of RGB and NIR Images Using Two Stage Convolutional Neural Networks. IEEE Access.

[79] Widjaja Putra B.T., Soni P. (2017). Evaluating NIR-Red and NIR-Red edge external filters with digital cameras for assessing vegetation indices under different illumination. Infrared Phys. Technol..

[80] Sakamoto T., Gitelson A.A., Nguy-Robertson A.L., Arkebauer T.J., Wardlow B.D., Suyker A.E., Verma S.B., Shibayama M. (2012). An alternative method using digital cameras for continuous monitoring of crop status. Agric. For. Meteorol..

[81] Zhang J., Yang C., Song H., Hoffmann W.C., Zhang D., Zhang G. (2016). Evaluation of an Airborne Remote Sensing Platform Consisting of Two Consumer-Grade Cameras for Crop Identification. Remote Sens..

[82] Wedding B. (2018). The Non-Invasive Assessment of Avocado Maturity and Quality. Ph.D. Thesis.

[83] Cozzolino D., Porker K., Laws M. (2015). An Overview on the Use of Infrared Sensors for in Field, Proximal and at Harvest Monitoring of Cereal Crops. Agriculture.

[84] Zhu Y., Cao P., Liu S., Zheng Y., Huang C. (2020). Development of a New Method for Turbidity Measurement Using Two NIR Digital Cameras. ACS Omega.

[85] Hunt E.R., Hively W.D., Fujikawa S.J., Linden D.S., Daughtry C.S.T., McCarty G.W. (2010). Acquisition of NIR-Green-Blue Digital Photographs from Unmanned Aircraft for Crop Monitoring. Remote Sens..

[86] Hunt E.R., Hively W.D., McCarty G.W., Daughtry C.S.T., Forrestal P.J., Kratochvil R.J., Carr J.L., Allen N.F., Fox-Rabinovitz J.R., Miller C.D. (2011). NIR-Green-Blue High-Resolution Digital Images for Assessment of Winter Cover Crop Biomass. GIScience Remote Sens..

[87] Bert G., Carolina B., Pilar G., Nicolaas T., Andy L. (2015). A tiny VIS-NIR snapshot multispectral camera. Advanced Fabrication Technologies for Micro/Nano Optics and Photonics VIII.

[88] Lebourgeois V., Bégué A., Labbé S., Mallavan B., Prévot L., Roux B. (2008). Can Commercial Digital Cameras Be Used as Multispectral Sensors? A Crop Monitoring Test. Sensors.

[89] Soria X., Sappa A.D., Akbarinia A. Multispectral single-sensor RGB-NIR imaging: New challenges and opportunities. Proceedings of the 2017 Seventh International Conference on Image Processing Theory, Tools and Applications (IPTA).

[90] Sahin H.M., Miftahushudur T., Grieve B., Yin H. (2023). Segmentation of weeds and crops using multispectral imaging and CRF-enhanced U-Net. Comput. Electron. Agric..

[91] Carbone C., Potena C., Nardi D., Obaidat M.S., Oren T., Rango F.D. (2022). Augmentation of Sunflower-Weed Segmentation Classification with Unity Generated Imagery Including Near Infrared Sensor Data. Simulation and Modeling Methodologies, Technologies and Applications.

[92] Yang C., Park B., Lu R. (2015). Hyperspectral Imagery for Mapping Crop Yield for Precision Agriculture. Hyperspectral Imaging Technology in Food and Agriculture.

[93] Komi P.J., Jackson M.R., Parkin R.M. Plant Classification Combining Colour and Spectral Cameras for Weed Control Purposes. Proceedings of the 2007 IEEE International Symposium on Industrial Electronics.

[94] Adão T., Hruška J., Pádua L., Bessa J., Peres E., Morais R., Sousa J.J. (2017). Hyperspectral Imaging: A Review on UAV-Based Sensors, Data Processing and Applications for Agriculture and Forestry. Remote Sens..

[95] Tallada J.G., Bato P.M., Shrestha B.P., Kobayashi T., Nagata M., Park B., Lu R. (2015). Quality Evaluation of Plant Products. Hyperspectral Imaging Technology in Food and Agriculture.

[96] Mahesh S., Jayas D.S., Paliwal J., White N.D.G. (2015). Hyperspectral imaging to classify and monitor quality of agricultural materials. J. Stored Prod. Res..

[97] Okamoto H., Murata T., Kataoka T., Hata S.-I. (2007). Plant classification for weed detection using hyperspectral imaging with wavelet analysis. Weed Biol. Manag..

[98] Lu R., Park B., Park B., Lu R. (2015). Introduction. Hyperspectral Imaging Technology in Food and Agriculture.

[99] Wendel A., Underwood J. Self-supervised weed detection in vegetable crops using ground based hyperspectral imaging. Proceedings of the 2016 IEEE International Conference on Robotics and Automation (ICRA).

[100] Kumar L., Schmidt K., Dury S., Skidmore A., Meer F.D.v.d., Jong S.M.D. (2001). Imaging Spectrometry and Vegetation Science. Imaging Spectrometry: Basic Principles and Prospective Applications.

[101] Kwon Noh H., Lu R. (2005). Hyperspectral Reflectance and Fluorescence for Assessing Apple Quality.

[102] Vrindts E., De Baerdemaeker J. Optical Weed Detection and Evaluation Using Reflection Measurements. Proceedings of the Photonics East (ISAM, VVDC, IEMB).

[103] Okamoto H., Murata T., Kataoka T., Hata S. WEED DETECTION USING HYPERSPECTRAL IMAGING. Automation Technology for Off-Road Equipment, Proceedings of the 7–8 October 2004 Conference (Kyoto, Japan).

[104] Caballero D., Calvini R., Amigo J.M., Amigo J.M. (2019). Chapter 3.3—Hyperspectral imaging in crop fields: Precision agriculture. Data Handling in Science and Technology.

[105] Feyaerts F., van Gool L. (2001). Multi-spectral vision system for weed detection. Pattern Recognit. Lett..

[106] Slaughter D.C., Giles D.K., Fennimore S.A., Smith R.F. (2008). Multispectral Machine Vision Identification of Lettuce and Weed Seedlings for Automated Weed Control. Weed Technol..

[107] Yu F., Jin Z., Guo S., Guo Z., Zhang H., Xu T., Chen C. (2022). Research on weed identification method in rice fields based on UAV remote sensing. Front. Plant Sci..

[108] Lee W.S., Park B., Lu R. (2015). Plant Health Detection and Monitoring. Hyperspectral Imaging Technology in Food and Agriculture.

[109] Vrindts E., De Baerdemaeker J., Ramon H. (2002). Weed Detection Using Canopy Reflection. Precis. Agric..

[110] Lee W.S., Slaughter D.C., Giles D.K. (1999). Robotic Weed Control System for Tomatoes. Precis. Agric..

[111] Su W.-H. (2020). Advanced Machine Learning in Point Spectroscopy, RGB-and Hyperspectral-Imaging for Automatic Discriminations of Crops and Weeds: A Review. Smart Cities.

[112] Middleton What Is Hyperspectral Imaging?. https://www.middletonspectral.com/resources/what-is-hyperspectral-imaging/.

[113] Liu B., Li R., Li H., You G., Yan S., Tong Q. (2019). Crop/Weed Discrimination Using a Field Imaging Spectrometer System. Sensors.

[114] Zwiggelaar R. (1998). A review of spectral properties of plants and their potential use for crop/weed discrimination in row-crops. Crop. Prot..

[115] Ravikanth L., Jayas D.S., White N.D.G., Fields P.G., Sun D.-W. (2017). Extraction of Spectral Information from Hyperspectral Data and Application of Hyperspectral Imaging for Food and Agricultural Products. Food Bioprocess Technol..

[116] Du J.-X., Wang X.-F., Zhang G.-J. (2007). Leaf shape based plant species recognition. Appl. Math. Comput..

[117] Furbank R.T., Tester M. (2011). Phenomics–technologies to relieve the phenotyping bottleneck. Trends Plant Sci..

[118] Jones H.G., Vaughan R.A. (2010). Remote Sensing of Vegetation: Principles, Techniques, and Applications.

[119] Jin J., Tang L. (2009). Corn plant sensing using real-time stereo vision. J. Field Robot..

[120] van der Veeken M., Tang L., Willem Hofstee J. (2006). Automated Corn Plant Spacing Measurement at Early Growth Stages Using Active Computer Vision.

[121] Weiss U., Biber P., Laible S., Bohlmann K., Zell A. Plant species classification using a 3D LIDAR sensor and machine learning. Proceedings of the 2010 Ninth International Conference on Machine Learning and Applications.

[122] Feyaerts F., Pollet P., Van Gool L., Wambacq P. (1999). Sensor for Weed Detection Based on Spectral Measurements. Proceedings of the Fourth International Conference on Precision Agriculture.

[123] Andujar D., Martinez-Guanter J. (2022). An Overview of Precision Weed Mapping and Management based on *Remote Sensing*. Remote Sens..

[124] Roberts J., Florentine S. (2024). Advancements and developments in the detection and control of invasive weeds: A global review of the current challenges and future opportunities. Weed Sci..

[125] Dale L.M., Thewis A., Boudry C., Rotar I., Dardenne P., Baeten V., Pierna J.A.F. (2013). Hyperspectral Imaging Applications in Agriculture and Agro-Food Product Quality and Safety Control: A Review. Appl. Spectrosc. Rev..

[126] Lu B., Dao P.D., Liu J., He Y., Shang J. (2020). Recent Advances of Hyperspectral Imaging Technology and Applications in Agriculture. Remote Sens..

[127] Tsoulias N., Zhao M., Paraforos D.S., Argyropoulos D., Zhang Q. (2022). Hyper- and Multi-spectral Imaging Technologies. Encyclopedia of Smart Agriculture Technologies.

[128] Che’Ya N.N., Dunwoody E., Gupta M. (2021). Assessment of Weed Classification Using Hyperspectral Reflectance and Optimal Multispectral UAV Imagery. Agronomy.

[129] de Castro A.-I., Jurado-Expósito M., Gómez-Casero M.-T., López-Granados F. (2012). Applying Neural Networks to Hyperspectral and Multispectral Field Data for Discrimination of Cruciferous Weeds in Winter Crops. Sci. World J..

[130] Manullang M.C., Lin Y.-H., Lai S.-J., Chou N.-K. (2021). Implementation of Thermal Camera for Non-Contact Physiological Measurement: A Systematic Review. Sensors.

[131] Leira F.S., Johansen T.A., Fossen T.I. Automatic detection, classification and tracking of objects in the ocean surface from UAVs using a thermal camera. Proceedings of the 2015 IEEE Aerospace Conference.

[132] Aaron E., Dharmendra S. (2019). Machine learning approaches to automate weed detection by UAV based sensors. Proc. SPIE.

[133] Wen T., Li J.-H., Wang Q., Gao Y.-Y., Hao G.-F., Song B.-A. (2023). Thermal imaging: The digital eye facilitates high-throughput phenotyping traits of plant growth and stress responses. Sci. Total Environ..

[134] Xu B., Meng R., Chen G., Liang L., Lv Z., Zhou L., Sun R., Zhao F., Yang W. (2023). Improved weed mapping in corn fields by combining UAV-based spectral, textural, structural, and thermal measurements. Pest Manag. Sci..

[135] Shirzadifar A., Bajwa S., Nowatzki J., Bazrafkan A. (2020). Field identification of weed species and glyphosate-resistant weeds using high resolution imagery in early growing season. Biosyst. Eng..

[136] Eide A., Koparan C., Zhang Y., Ostlie M., Howatt K., Sun X. (2021). UAV-Assisted Thermal Infrared and Multispectral Imaging of Weed Canopies for Glyphosate Resistance Detection. Remote Sens..

[137] Pineda M., Barón M., Pérez-Bueno M.-L. (2021). Thermal Imaging for Plant Stress Detection and Phenotyping. Remote Sens..

[138] Peteinatos G.G., Weis M., Andújar D., Rueda Ayala V., Gerhards R. (2014). Potential use of ground-based sensor technologies for weed detection. Pest Manag. Sci..

[139] Machleb J., Peteinatos G.G., Kollenda B.L., Andújar D., Gerhards R. (2020). Sensor-based mechanical weed control: Present state and prospects. Comput. Electron. Agric..

[140] Brown R.B., Noble S.D. (2005). Site-specific weed management: Sensing requirements—what do we need to see?. Weed Sci..

[141] Raja R., Nguyen T.T., Slaughter D.C., Fennimore S.A. (2020). Real-time robotic weed knife control system for tomato and lettuce based on geometric appearance of plant labels. Biosyst. Eng..

[142] Nanni M.R., Demattê J.A.M., Rodrigues M., dos Santos G.L.A.A., Reis A.S., de Oliveira K.M., Cezar E., Furlanetto R.H., Crusiol L.G.T., Sun L. (2021). Mapping Particle Size and Soil Organic Matter in Tropical Soil Based on Hyperspectral Imaging and Non-Imaging Sensors. Remote Sens..

[143] Åstrand B., Baerveldt A.-J. (2002). An Agricultural Mobile Robot with Vision-Based Perception for Mechanical Weed Control. Auton. Robot..

[144] Klose R., Marquering D.J., Thiel M., Ruckelshausen A., Strautmann B. (2008). Weedy—A Sensor Fusion Based Autonomous Field Robot for Selective Weed Control. https://www.semanticscholar.org/paper/Weedy-%E2%80%93-a-sensor-fusion-based-autonomous-field-for-Klose-Marquering/727ae9cd0ef2ee4741479210ad39dc496ad95b0b.

[145] Pire T., Mujica M., Civera J., Kofman E. (2019). The Rosario dataset: Multisensor data for localization and mapping in agricultural environments. Int. J. Robot. Res..

[146] Fountas S., Mylonas N., Malounas I., Rodias E., Hellmann Santos C., Pekkeriet E. (2020). Agricultural Robotics for Field Operations. Sensors.

[147] Atefi A., Ge Y., Pitla S., Schnable J. (2021). Robotic Technologies for High-Throughput Plant Phenotyping: Contemporary Reviews and Future Perspectives. Front. Plant Sci..

[148] Andreasen C., Rakhmatulin I., Saberi M., Zhang Z. (2024). Weed control with laser beams: An eco-friendly alternative to herbicides and mechanical weed control. AIP Conf. Proc..

[149] Andreasen C., Vlassi E., Salehan N., Johannsen K.S., Jensen S.M. (2024). Laser weed seed control: Challenges and opportunities. Front. Agron..

[150] Sahba K., Askraba S., Alameh K.E. (2006). Non-contact laser spectroscopy for plant discrimination in terrestrial crop spraying. Opt. Express.

[151] Bloomer D.J., Harrington K.C., Ghanizadeh H., James T.K. (2024). Robots and shocks: Emerging non-herbicide weed control options for vegetable and arable cropping. N. Z. J. Agric. Res..

[152] Mathiassen S.K., Bak T., Christensen S., Kudsk P. (2006). The Effect of Laser Treatment as a Weed Control Method. Biosyst. Eng..

[153] Zhu H., Zhang Y., Mu D., Bai L., Zhuang H., Li H. (2022). YOLOX-based blue laser weeding robot in corn field. Front. Plant Sci..

[154] Fatima H.S., ul Hassan I., Hasan S., Khurram M., Stricker D., Afzal M.Z. (2023). Formation of a Lightweight, Deep Learning-Based Weed Detection System for a Commercial Autonomous Laser Weeding Robot. Appl. Sci..

[155] Reiser D., Sehsah E.-S., Bumann O., Morhard J., Griepentrog H.W. (2019). Development of an Autonomous Electric Robot Implement for Intra-Row Weeding in Vineyards. Agriculture.

[156] Chen Z.J., Shan K.L., Guo Q.Y. (2012). Laser Navigation System Applied to Weeding Robot. Appl. Mech. Mater..

[157] Andreasen C., Scholle K., Saberi M. (2022). Laser Weeding With Small Autonomous Vehicles: Friends or Foes?. Front. Agron..

[158] Rakhmatulin I., Andreasen C. (2020). A Concept of a Compact and Inexpensive Device for Controlling Weeds with Laser Beams. In Agronomy.

[159] Krupanek J., de Santos P.G., Emmi L., Wollweber M., Sandmann H., Scholle K., Di Minh Tran D., Schouteten J.J., Andreasen C. (2024). Environmental performance of an autonomous laser weeding robot—A case study. Int. J. Life Cycle Assess.

[160] Andreasen C., Vlassi E., Salehan N. (2024). Laser weeding: Opportunities and challenges for couch grass (*Elymus repens* (L.) Gould) control. Sci. Rep..

[161] Wang M., Leal-Naranjo J.A., Ceccarelli M., Blackmore S. (2022). A Novel Two-Degree-of-Freedom Gimbal for Dynamic Laser Weeding: Design, Analysis, and Experimentation. IEEE/ASME Trans. Mechatron..

[162] Dille J.A., Milner M., Groeteke J.J., Mortensen D.A., Williams M.M. (2003). How good is your weed map? A comparison of spatial interpolators. Weed Sci..

[163] Upadhyaya S., Ehsani M., Mattson M.L. (2003). Method and Apparatus for Ultra Precise GPS-Based Mapping of Seeds or Vegetation during Planting. Google Patents.

[164] Krähmer H., Andreasen C., Economou-Antonaka G., Holec J., Kalivas D., Kolářová M., Novák R., Panozzo S., Pinke G., Salonen J. (2020). Weed surveys and weed mapping in Europe: State of the art and future tasks. Crop. Prot..

[165] Ehsani M., Upadhyaya S., Mattson M. (2004). Seed location mapping using RTK GPS. Trans. ASAE.

[166] Griepentrog H.W., Nørremark M., Nielsen H., Blackmore B.S. (2005). Seed Mapping of Sugar Beet. Precis. Agric..

[167] Abidine A.Z., Heidman B.C., Upadhyaya S.K., Hills D.J. (2004). Autoguidance system operated at high speed causes almost no tomato damage. Calif. Agric..

[168] Pflanz M., Nordmeyer H., Schirrmann M. (2018). Weed Mapping with UAS Imagery and a Bag of Visual Words Based Image Classifier. Remote Sens..

[169] LÓPez-Granados F. (2011). Weed detection for site-specific weed management: Mapping and real-time approaches. Weed Res..

[170] Kanagasingham S., Ekpanyapong M., Chaihan R. (2020). Integrating machine vision-based row guidance with GPS and compass-based routing to achieve autonomous navigation for a rice field weeding robot. Precis. Agric..

[171] DFROBOT How RTK Technology Enhances Robotic Lawn Mower Precision. https://www.dfrobot.com/blog-13511.html.

[172] Andújar D., Escolà A., Rosell-Polo J.R., Fernández-Quintanilla C., Dorado J. (2013). Potential of a terrestrial LiDAR-based system to characterise weed vegetation in maize crops. Comput. Electron. Agric..

[173] Debnath S., Paul M., Debnath T. (2023). Applications of LiDAR in Agriculture and Future Research Directions. J. Imaging.

[174] Rivera G., Porras R., Florencia R., Sánchez-Solís J.P. (2023). LiDAR applications in precision agriculture for cultivating crops: A review of recent advances. Comput. Electron. Agric..

[175] Shahbazi N., Ashworth M.B., Callow J.N., Mian A., Beckie H.J., Speidel S., Nicholls E., Flower K.C. (2021). Assessing the Capability and Potential of LiDAR for Weed Detection. Sensors.

[176] Andújar D., Rueda-Ayala V., Moreno H., Rosell-Polo J.R., Escolá A., Valero C., Gerhards R., Fernández-Quintanilla C., Dorado J., Griepentrog H.-W. (2013). Discriminating Crop, Weeds and Soil Surface with a Terrestrial LIDAR Sensor. Sensors.

[177] Fernández-Quintanilla C., Peña J.M., Andújar D., Dorado J., Ribeiro A., López-Granados F. (2018). Is the current state of the art of weed monitoring suitable for site-specific weed management in arable crops?. Weed Res..

[178] Fuchs S., Hirzinger G. Extrinsic and depth calibration of ToF-cameras. Proceedings of the 2008 IEEE Conference on Computer Vision and Pattern Recognition.

[179] Gai J., Xiang L., Tang L. (2021). Using a depth camera for crop row detection and mapping for under-canopy navigation of agricultural robotic vehicle. Comput. Electron. Agric..

[180] Nakarmi A.D., Tang L. (2012). Automatic inter-plant spacing sensing at early growth stages using a 3D vision sensor. Comput. Electron. Agric..

[181] Toa M., Whitehead A. (2020). Ultrasonic Sensing Basics.

[182] Andújar D., Weis M., Gerhards R. (2012). An Ultrasonic System for Weed Detection in Cereal Crops. Sensors.

[183] Chandra Swain K., Uz Zaman Q., W Schumann A., C Percival D. (2009). Detecting Weed and Bare-spot in Wild Blueberry Using Ultrasonic Sensor Technology.

[184] Amid H., Desa A., Ibrahim A.H., Redmond Ramin S., Siva K.B., Muhammad Y., Jun Z., Baohua Z. (2018). Development of a Field Robot Platform for Mechanical Weed Control in Greenhouse Cultivation of Cucumber. Agricultural Robots.

[185] Rohani A. (2022). Weed detection using ultrasonic signal processing employing artificial neural network (ANN) with efficient extracted features. Agric. Eng. Int. CIGR J..

[186] Li Y., Guo Z., Shuang F., Zhang M., Li X. (2022). Key technologies of machine vision for weeding robots: A review and benchmark. Comput. Electron. Agric..

[187] Shi J., Bai Y., Diao Z., Zhou J., Yao X., Zhang B. (2023). Row Detection BASED Navigation and Guidance for Agricultural Robots and Autonomous Vehicles in Row-Crop Fields: Methods and Applications. Agronomy.

[188] Zhang Y., Wang M., Zhao D., Liu C., Liu Z. (2023). Early weed identification based on deep learning: A review. Smart Agric. Technol..

[189] Hasan A.S.M.M., Sohel F., Diepeveen D., Laga H., Jones M.G.K. (2021). A survey of deep learning techniques for weed detection from images. Comput. Electron. Agric..

[190] Albiero D., Pontin Garcia A., Kiyoshi Umezu C., Leme de Paulo R. (2022). Swarm robots in mechanized agricultural operations: A review about challenges for research. Comput. Electron. Agric..

[191] Alatise M.B., Hancke G.P. (2020). A Review on Challenges of Autonomous Mobile Robot and Sensor Fusion Methods. IEEE Access.

[192] Qu J., Zhang Z., Qin Z., Guo K., Li D. (2024). Applications of Autonomous Navigation Technologies for Unmanned Agricultural Tractors: A Review. Machines.

[193] Singh V., Rana A., Bishop M., Filippi A.M., Cope D., Rajan N., Bagavathiannan M., Sparks D.L. (2020). Chapter Three—Unmanned aircraft systems for precision weed detection and management: Prospects and challenges. Advances in Agronomy.

[194] Dhanya V.G., Subeesh A., Kushwaha N.L., Vishwakarma D.K., Nagesh Kumar T., Ritika G., Singh A.N. (2022). Deep learning based computer vision approaches for smart agricultural applications. Artif. Intell. Agric..

[195] Rao B.B.P., Saluia P., Sharma N., Mittal A., Sharma S.V. Cloud computing for Internet of Things & sensing based applications. Proceedings of the 2012 Sixth International Conference on Sensing Technology (ICST).

[196] Javaid M., Haleem A., Singh R.P., Suman R. (2022). Enhancing smart farming through the applications of Agriculture 4.0 technologies. Int. J. Intell. Netw..

[197] Edan Y., Adamides G., Oberti R., Nof S.Y. (2023). Agriculture Automation. Springer Handbook of Automation.

[198] Aravind K.R., Raja P., Pérez-Ruiz M. (2017). Task-based agricultural mobile robots in arable farming: A review. Span. J. Agric. Res..

[199] Shaner D.L., Beckie H.J. (2014). The future for weed control and technology. Pest Manag. Sci..

[200] Araus J.L., Cairns J.E. (2014). Field high-throughput phenotyping: The new crop breeding frontier. Trends Plant Sci..

[201] Ball D., Ross P., English A., Milani P., Richards D., Bate A., Upcroft B., Wyeth G., Corke P. (2017). Farm Workers of the Future: Vision-Based Robotics for Broad-Acre Agriculture. IEEE Robot. Autom. Mag..

[202] Epée Missé P.T., Werner A., Almond P. (2020). Developing automated and autonomous weed control methods on vegetable crops in New Zealand. SSRN.

[203] Schmitz A., Badgujar C., Mansur H., Flippo D., McCornack B., Sharda A. (2022). Design of a Reconfigurable Crop Scouting Vehicle for Row Crop Navigation: A Proof-of-Concept Study. Sensors.

[204] Chostner B. (2017). See & spray: The next generation of weed control. Resour. Mag..

[205] Vasconcelos G.J.Q., Costa G.S.R., Spina T.V., Pedrini H. (2023). Low-Cost Robot for Agricultural Image Data Acquisition. Agriculture.

[206] Bangale R., Kumar M. (2024). Robot-Based Weed Identification and Control System. Precision Agriculture for Sustainability.

[207] Redbond M. (2015). Robots-the future of agriculture. Int. Pest Control.

[208] Das T., Sen S., Ghosh S., Paramanik B., Dattaand D., Roy A. (2021). Integrated Weed Management in Crops and Cropping Systems: Concept, Needs and Challenges. New Delhi Publ..

[209] Paulus S., Behmann J., Mahlein A.-K., Plümer L., Kuhlmann H. (2014). Low-Cost 3D Systems: Suitable Tools for Plant Phenotyping. Sensors.

[210] Alchanatis V., Cohen A., Cohen Y., Levi O., Naor A. Multimodal remote sensing for enhancing detection of spatial variability in agricultural fields. Proceedings of the Spatial2 Conference: Spatial Data Methods for Environmental and Ecological Processes.

[211] Duckett T., Pearson S., Blackmore S., Grieve B., Chen W.-H., Cielniak G., Cleaversmith J., Dai J., Davis S., Fox C. (2018). Agricultural robotics: The future of robotic agriculture. arXiv.

[212] Gómez-Candón D. (2020). Remote imagery to assess water stress variability within the orchard. CABI Rev..

